# Laminin switches terminal differentiation fate of human trophoblast stem cells under chemically defined culture conditions

**DOI:** 10.1016/j.jbc.2023.104650

**Published:** 2023-03-25

**Authors:** Victoria Karakis, Mahe Jabeen, John W. Britt, Abigail Cordiner, Adam Mischler, Feng Li, Adriana San Miguel, Balaji M. Rao

**Affiliations:** 1 Department of Chemical and Biomolecular Engineering, North Carolina State University, Raleigh, North Carolina, USA; 2 Department of Genetics, North Carolina State University, Raleigh, North Carolina, USA; 3 Department of Pathology and Laboratory Medicine, University of North Carolina-Chapel Hill, Chapel Hill, North Carolina, USA; 4 Golden LEAF Biomanufacturing Training and Education Center, North Carolina State University, Raleigh, North Carolina, USA

**Keywords:** trophoblast, trophoblast stem cells, placenta, extravillous trophoblast, syncytiotrophoblast, differentiation

## Abstract

Human trophoblast stem cells (hTSCs) have emerged as a powerful tool to model early placental development *in vitro*. Analogous to the epithelial cytotrophoblast in the placenta, hTSCs can differentiate into cells of the extravillous trophoblast (EVT) lineage or the multinucleate syncytiotrophoblast (STB). Here we present a chemically defined culture system for STB and EVT differentiation of hTSCs. Notably, in contrast to current approaches, we neither utilize forskolin for STB formation nor transforming growth factor-beta (TGFβ) inhibitors or a passage step for EVT differentiation. Strikingly, the presence of a single additional extracellular cue–laminin-111–switched the terminal differentiation of hTSCs from STB to the EVT lineage under these conditions. In the absence of laminin-111, STB formation occurred, with cell fusion comparable to that obtained with differentiation mediated by forskolin; however, in the presence of laminin-111, hTSCs differentiated to the EVT lineage. Protein expression of nuclear hypoxia-inducible factors (HIF1α and HIF2α) was upregulated during EVT differentiation mediated by laminin-111 exposure. A heterogeneous mixture of Notch1^+^ EVTs in colonies and HLA-G^+^ single-cell EVTs were obtained without a passage step, reminiscent of heterogeneity *in vivo*. Further analysis showed that inhibition of TGFβ signaling affected both STB and EVT differentiation mediated by laminin-111 exposure. TGFβ inhibition during EVT differentiation resulted in decreased HLA-G expression and increased Notch1 expression. On the other hand, TGFβ inhibition prevented STB formation. The chemically defined culture system for hTSC differentiation established herein facilitates quantitative analysis of heterogeneity that arises during hTSC differentiation and will enable mechanistic studies *in vitro*.

The placenta is a complex fetal organ with a vast network of villi that ensures efficient exchange of nutrients and waste across the maternal-fetal interface. Epithelial cytotrophoblasts (CTBs) of the early human placenta give rise to all trophoblast cell types in the placenta (1, 2, 3, 4). CTBs undergo cell fusion to form the multinucleate syncytiotrophoblast (STB) that overlays the CTB layer of placental villi (1, 3). The STB is subsequently bathed in maternal blood at ∼10 weeks of gestation, when blood flow to the placenta is established (5, 6, 7). CTBs of placental villi anchored to the maternal decidua push through the syncytial layer and differentiate to extravillous trophoblasts (EVTs), first forming proliferative column trophoblasts adjacent to the villus tip (1, 3). At the distal end, column trophoblasts undergo an epithelial-to-mesenchymal transition (EMT) to become mature mesenchymal EVTs that invade the maternal decidua and parts of the myometrium (8, 9, 10, 11). These invasive trophoblasts aid in remodeling maternal spiral arteries and play a critical role in establishing sufficient perfusion of the placenta with maternal blood (12, 13, 14, 15).

Many pregnancy complications including preeclampsia, fetal growth restriction, miscarriage, and stillbirth are often a result of impaired arterial remodeling and evidence points to improper EVT differentiation and invasion as a major contributor to these pathologies (3, 16, 17, 18, 19, 20). For instance, quantitative analyses of images from preeclamptic placenta biopsies demonstrate shallow trophoblast invasion compared with healthy placenta (21). Previous studies have also suggested that increased apoptosis and an inability of CTBs to differentiate toward the EVT lineage may underlie shallow trophoblast invasion in preeclampsia (22, 23). Yet, despite the importance of EVT differentiation in placental health and pathology, molecular mechanisms underlying EVT differentiation and maturation to mesenchymal invasive EVTs remain poorly understood.

Mechanistic studies on early human placental development have been impeded primarily due to lack of suitable model systems. There are substantial restrictions on research with fetal tissue and human embryos and significant differences between placental development in common experimental animals and humans (24, 25, 26, 27, 28, 29). Additionally, there are significant differences in the transcriptome profiles of immortalized cell lines and primary trophoblasts (30). Further, immortalized cell lines do not model the heterogeneity of cell types observed during trophoblast differentiation *in vivo* (31). In this context, human trophoblast stem cells (hTSCs) derived from early gestation primary placental samples and blastocysts have emerged as powerful *in vitro* models of early human placental development (32). hTSCs, which model the CTB *in vivo*, can be maintained in cell culture and differentiate into STB or EVTs. In recent work, others and we have also shown that hTSCs can be derived from human pluripotent stem cells, including induced pluripotent stem cells (33, 34, 35, 36, 37, 38, 39, 40, 41) and villous cytotrophoblasts from term placentas (42), raising the exciting prospect of investigating pathological trophoblast development using somatic tissues (*e.g.*, placenta) obtained at birth (42, 43). Nevertheless, current protocols for hTSC differentiation to EVT and STB limit mechanistic studies.

Most current protocols for *in vitro* differentiation of hTSCs include transforming growth factor-beta (TGFβ) inhibitors during EVT differentiation (32, 44, 45, 46, 47). However, this is problematic because TGFβ plays an important role in EVT differentiation and dysregulation of TGFβ signaling can result in placental pathologies (11, 17, 20, 48, 49, 50, 51, 52, 53, 54). Inhibition of TGFβ during EVT differentiation precludes investigation into the role of TGFβ signaling during normal or pathological trophoblast differentiation. Similarly, STB differentiation *in vitro* predominantly relies on the use of forskolin to induce cell fusion. Forskolin raises cyclic AMP concentrations causing upregulation of GCM1, which controls expression of fusion genes, syncytin-1 and syncytin-2 (55). The use of forskolin impedes studies on extracellular cues regulating STB differentiation. Finally, it is important to note that heterogeneity of cell types is a central feature of EVT differentiation *in vivo*. Initially, epithelial CTBs form proliferative proximal cell columns that express column markers MYC, VE-cadherin, Notch1, as well as CTB markers EGFR and ITGA6 (3, 8, 11, 32, 50, 56, 57, 58, 59, 60, 61, 62, 63, 64). Differentiating EVTs at the distal end of the epithelial column remain proliferative; however, they begin to express ITGA5 and low levels of the mature EVT marker HLA-G and lose CTB marker expression (11, 57, 62, 65). Subsequently, when these distal EVTs undergo EMT, they gain expression of ITGA1, express higher levels of HLA-G, and lose expression of markers that characterize the column trophoblasts (3, 66, 67). Current protocols for EVT differentiation that include a passage step (32, 47) do not capture EVT heterogeneity and the sequential nature of CTB differentiation as mature mesenchymal EVTs are formed.

Here we present chemically defined culture conditions for differentiation of placenta- and hiPSC-derived hTSCs to EVTs and STB. Notably, our conditions do not involve a passage step and exclude forskolin and TGFβ inhibition during STB and EVT differentiation, respectively. Under these culture conditions, we identified laminin-111-mediated upregulation of hypoxia-inducible factor-alpha (HIFα) as the critical input that switches differentiation hTSCs from STB to the EVT lineage. We also investigated the effect of inhibiting TGFβ signaling on EVT and STB differentiation.

## Results

### Chemically defined conditions for STB differentiation in the absence of forskolin

**Figure 1:**
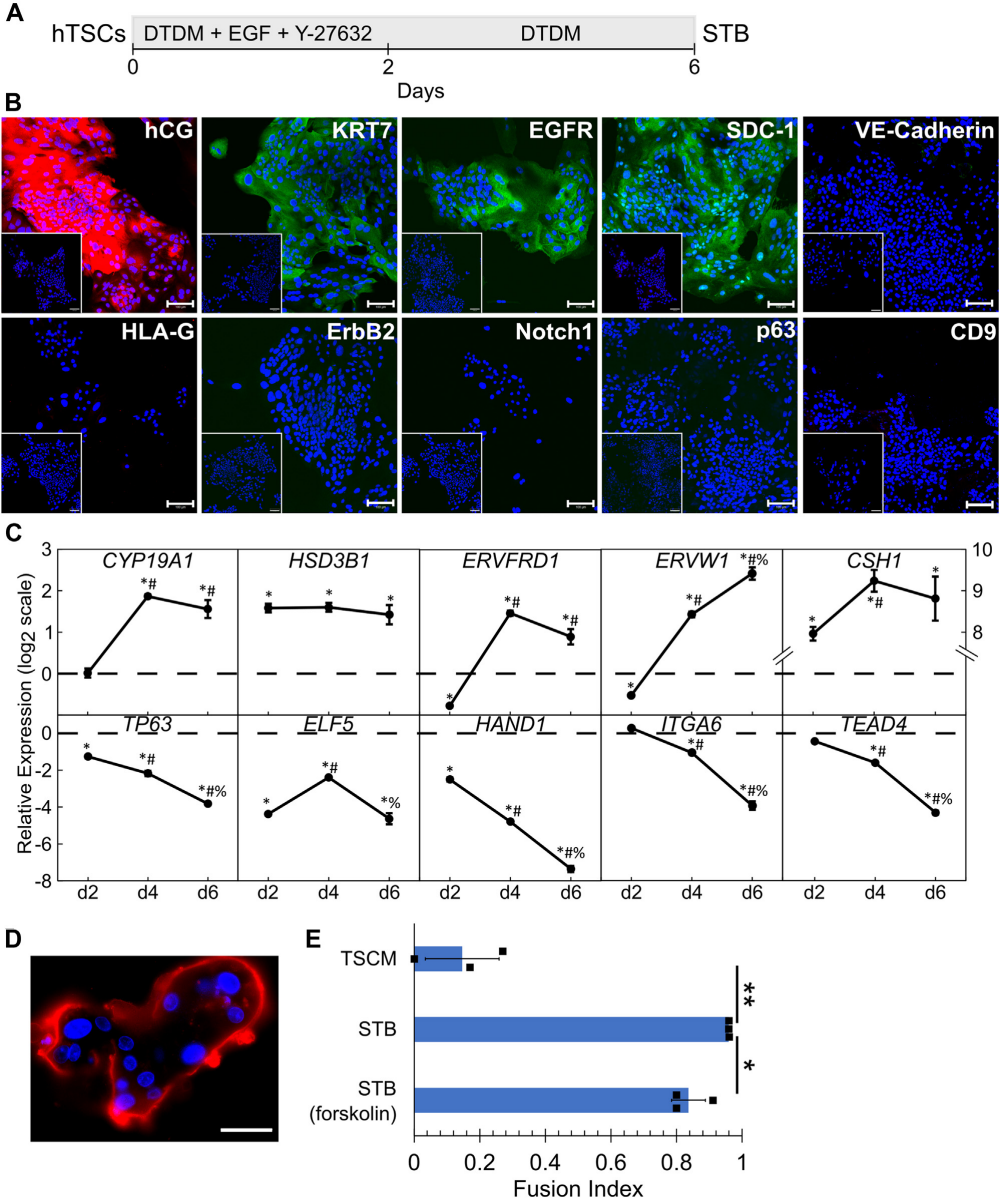
**Chemically defined conditions for STB differentiation in the absence of forskolin.***A*, schematic of protocol for hTSC differentiation to STB. *B*, confocal images of CT30 hTSCs on day 6 of STB differentiation, staining for hCG, KRT7, EGFR, SDC-1, VE-Cadherin, HLA-G, ErbB2, Notch1, p63, and CD9. Nuclei were stained with DAPI. Inset is the respective isotype control. *C*, gene expression of *CYP19A1*, *HSD3B1*, *ERVFRD1*, *ERVW1*, *CSH1*, *TP63*, *ELF5*, *HAND1*, *ITGA6*, and *TEAD4* of CT30 hTSCs on day 2, day 4, and day 6 of STB differentiation compared to undifferentiated hTSCs (*dashed line*). Three biological replicates were used (Error bars, S.E., ∗*p* < 0.05 for comparison with undifferentiated hTSCs, ^#^*p* < 0.05 for comparison with cells at day 2, ^%^*p* < 0.05 for comparison with cells at day 4). *D*, fluorescent image of CT30 hTSCs on day 6 of STB differentiation. Nuclei were stained with DAPI. Membrane was stained with Di-8-ANEPPS cell membrane stain. The scale bar represents 50 μm. *E*, fusion efficiency of CT30 hTSCs on day 6 of STB differentiation using the method described in Panel *A* and the method using forskolin as previously described (32) compared to CT30 hTSCs cultured in TSCM. Fusion index is calculated as (N-S)/T where N is the number of nuclei in the syncytia, S is the number of syncytia, and T is the total number of nuclei counted. Nuclei were stained with DAPI. Membrane was stained with Di-8-ANEPPS cell membrane stain. Three measurements from two biological replicates were used to calculate fusion index (∗*p* < 0.05, ∗∗*p* < 0.005, Error bars, S.D., n = 3). The scale bars represent 100 μm for all images unless specified otherwise. hTSC, human trophoblast stem cell; STB, syncytiotrophoblast; TSCM, trophoblast stem cell medium.

Placenta-derived CT29 and CT30 hTSCs and hiPSC-derived SC102A-1 hTSCs were cultured in trophoblast stem cell medium (TSCM) as described previously (32, 38). Differentiation was induced by passaging hTSCs into a defined trophoblast differentiation medium (DTDM) supplemented with epidermal growth factor (EGF) and the ROCK inhibitor, Y-27632, at passage for 2 days, and culturing them for an additional 4 days in DTDM (Fig. 1*A*). Upon passage, we initially observed an increase in cell number, but by day 6, cells in a flat monolayer without visible cell boundaries were observed; lacunae were also detected (Fig. S1*A*). Immunofluorescence revealed expression of STB markers, hCG, SDC-1, as well as the pan-trophoblast marker, KRT7 on day 6 (Figs. 1*B* and S1, *B* and *D*). Differentiated cells also expressed EGH receptor, which has been previously seen in villous cytotrophoblasts, STB, and proximal column EVTs (EGFR; Figs. 1*B* and S1, *B* and *D*) (68). EVT markers, HLA-G, Notch1, ErbB2, VE-Cadherin, or CD9, as well as the CTB marker, p63, were not expressed, suggesting that EVT differentiation did not occur under these conditions and that complete differentiation to STB occurred (Fig. 1*B* and S1, *B* and *D*). High-throughput qPCR analysis revealed that STB genes *CYP19A1*, *HSD3B1*, *ERVFRD1*, *ERVW1*, and *CSH1* were all upregulated by day 4 of STB differentiation in CT30 hTSCs and continued to be overexpressed on day 6 of differentiation compared to undifferentiated hTSCs at day 0 (Fig. 1*C*). The comprehensive dataset from qPCR analysis is included in Tables S1 and S2. Similarly, in CT29 hTSCs, *HSD3B1*, *ERVFRD1*, and *CSH1* were upregulated by day 4 of STB differentiation compared with hTSCs at day 0, in addition to other STB markers *CGB*, *GCM1*, and *SDC1* (Fig. S1*F*). Additionally, we saw evidence of increasing STB marker expression over the 6-days differentiation period. For example, *CYP19A1* and *ERVW1* were upregulated on day 4 compared to day 2 of differentiation in CT29 hTSCs and *ERVW1* in CT30 hTSCs and *CGB* in CT29 hTSCs were upregulated by day 6 of differentiation compared to day 4 (Fig. 1*C* and S1*F*). On the other hand, CTB genes were downregulated over the 6-days differentiation period. Specifically, in CT30 hTSCs, *TP63*, *ELF5*, and *HAND1* were downregulated by day 2 of STB differentiation and *ITGA6* and *TEAD4* were downregulated by day 4 of STB differentiation compared to day 0 (Fig. 1*C*). *TP63*, *HAND1*, *ITGA6*, and *TEAD4* all had a significant reduction in expression on day 4 compared to day 2 of differentiation and on day 6 compared to day 4 of differentiation (Fig. 1*C*). In CT29 hTSCs, *TP63*, *ELF5*, *HAND1*, *ITGA6*, *TEAD4*, *CDH1* (E-Cadherin), and *YAP* were all downregulated by day 6 of STB differentiation with respect to day 0 (Fig. S1*F*). Lastly, a membrane stain revealed multinucleate cells with a fusion index higher than that obtained during STB differentiation using a protocol described by Okae *et al.* (32) where forskolin is used (Figs. 1, *D* and *E* and S1, *C* and *E*). These results show that efficient STB differentiation of hTSCs occurs under our chemically defined conditions, in the absence of forskolin.

### Presence of laminin-111 switches hTSC differentiation from STB to EVT fate

**Figure 2:**
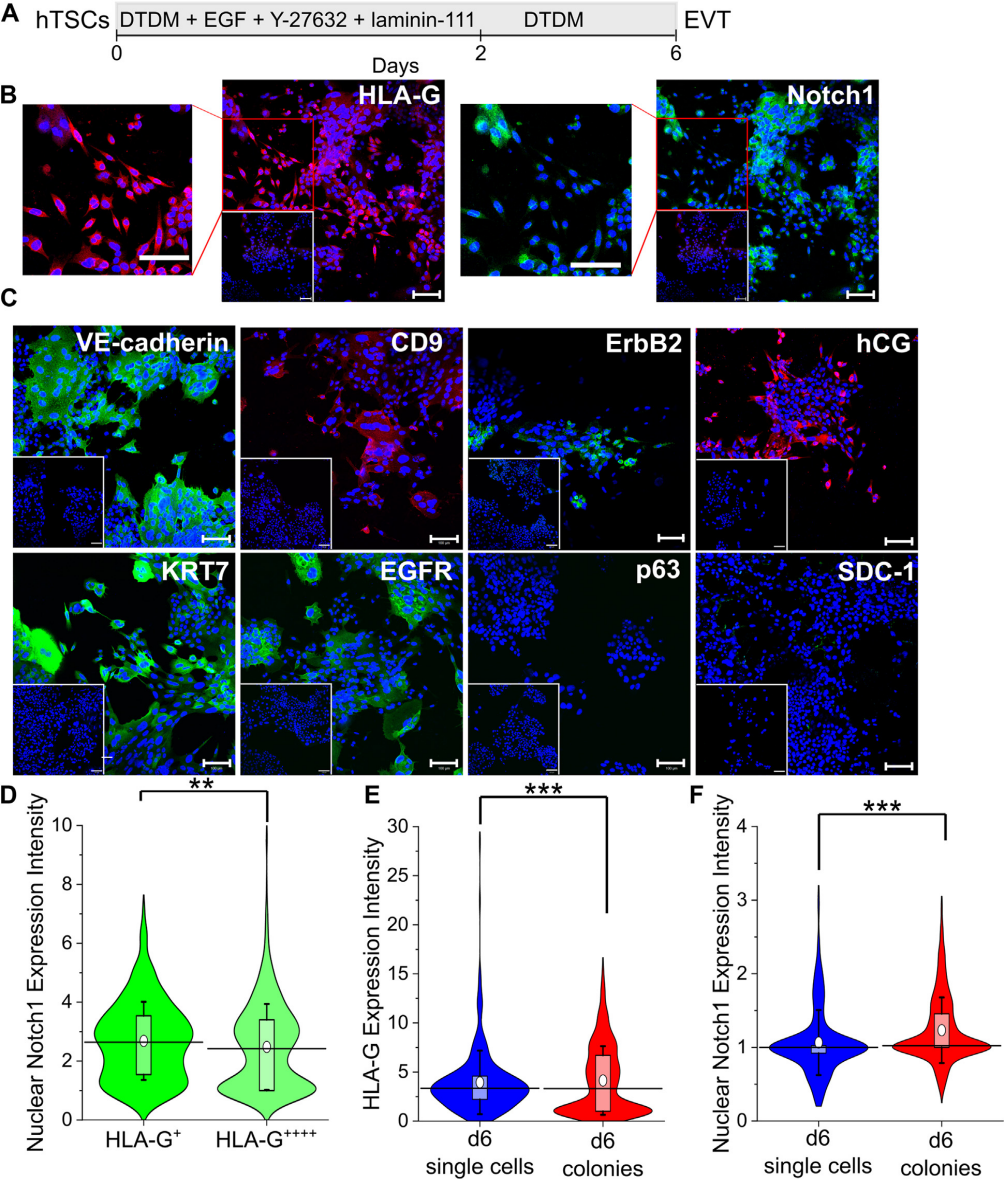
**Presence of laminin-111 switches hTSC differentiation from STB to EVT fate.***A*, schematic of protocol for hTSC differentiation to EVT. *B*, confocal images of CT30 hTSCs on day 6 of EVT differentiation, staining for HLA-G and Notch1. Nuclei were stained with DAPI. Inset is the respective isotype control. Outcrop is the magnified image. *C*, confocal images of CT30 hTSCs on day 6 of EVT differentiation, staining for VE-cadherin, CD9, ErbB2, KRT7, EGFR, p63, hCG, and SDC-1. Nuclei were stained with DAPI. Inset is the respective isotype control. *D*, quantitative analysis of Notch1 expression intensity of CT30 hTSCs on day 6 of EVT differentiation from the *bottom* (HLA-G^+^) and *top* (HLA-G^++++^) 25% of HLA-G expression intensity cells (n = 534, each). Analysis was performed in MATLAB and 2 biological replicates were used. The *white circle* represents the mean and the *black bar* represents the median (∗∗*p* < 0.005). *E*, quantitative analysis of HLA-G expression intensity of CT30 hTSCs on day 6 of EVT differentiation grouped into two categories: cells with no neighboring cells within a radius of 50 μm (n = 248) labeled as d6 single cells or cells with at least one or more neighboring cells within a 50 μm radius (n = 1061) labeled as d6 colonies. Analysis was performed in MATLAB and two biological replicates were used. The *white circle* represents the mean and the *black bar* represents the median (∗∗∗*p* < 0.0005). *F*, quantitative analysis of Notch1 expression intensity of CT30 hTSCs on day 6 of EVT differentiation grouped into two categories: cells with no neighboring cells within a radius of 50 μm (n = 300) labeled as d6 single cells or cells with at least one or more neighboring cells within a 50 μm radius (n = 1428) labeled as d6 colonies. Analysis was performed in MATLAB and two biological replicates were used. The *white circle* represents the mean and the *black bar* represents the median (∗∗∗*p* < 0.0005). The scale bars represent 100 μm for all images. EVT, extravillous trophoblast; hTSC, human trophoblast stem cell; STB, syncytiotrophoblast.

The protocol described by Okae *et al.* (32) for EVT differentiation utilizes Matrigel and absence of Matrigel results in the formation of both EVT-like and STB-like cells. Since laminin-111 is a major component of Matrigel, we hypothesized that laminin-111 may mediate EVT differentiation of hTSCs (69). Accordingly, we differentiated hTSCs using the previously described STB differentiation protocol (Fig. 1*A*), with the addition of laminin-111 for 2 days following passage and further culture for an additional 4 days in DTDM (Fig. 2*A*). Addition of laminin-111 resulted in a thin monolayer of matrix that solidified on the plate, covering the cells underneath (Fig. S2*A*). Upon initiation of differentiation, we observed that cells initially proliferated to form epithelial colonies. However, by day 6 of differentiation, single mesenchymal cells could be observed (Figs. 2*B* and S2, *B*, *C* and *G*); this is reminiscent of an epithelial to mesenchymal transition *in vivo*, where mature mesenchymal EVTs forming from the distal end of trophoblast columns *in vivo* (67). Differentiated cells expressed the EVT markers HLA-G, Notch1, EGFR, VE-cadherin, CD9, ErbB2, and the pan-trophoblast marker KRT7; however, the CTB marker p63 was not expressed (Figs. 2, *B* and *C* and S2, *C* and *G*). Additionally, we did not observe expression of the STB marker, SDC-1 (Figs. 2*C* and S2*C*). Importantly, *in vivo*, EVTs in the proximal column express Notch1 and EGFR and lower levels of HLA-G whereas the later-stage mesenchymal EVT express ErbB2 and HLA-G to a much greater extent and lose expression of Notch1 (3, 11, 57, 58, 59, 62, 68). It has also been previously observed that late-stage invasive EVTs along with STB express hCG, but it is absent from early, proliferative EVTs (70, 71). We observed this variation in HLA-G, Notch1, EGFR, ErbB2, and hCG expression in immunofluorescence images (Figs. 2, *B* and *C* and S2, *C* and *G*). Specifically, cells within colonies had low HLA-G and did not express ErbB2 nor hCG whereas single cells exhibited high HLA-G, ErbB2, and hCG expression. To further investigate marker localization, we used quantitative image analysis on HLA-G and Notch1 costained immunofluorescent images. Quantitative analysis revealed that cells in the bottom quartile of HLA-G expression (HLA-G^+^ *i.e.*, 25% of total cells that showed the lowest HLA-G expression intensity) indeed expressed Notch1 with a greater intensity than cells that were of the top HLA-G expression quartile (HLA-G^++++^ *i.e.*, 25% of total cells that showed the highest HLA-G expression intensity) in day 6 EVTs derived from CT29 and CT30 hTSCs; however, the increase in Notch1 intensity for HLA-G^+^ cells was not statistically significant in SC102A-1 hTSCs (Figs. 2*D* and S2, *D* and *H*). Analogous to EVTs *in vivo*, cells in the epithelial colonies appeared to express higher levels of Notch1 and lower levels of HLA-G than the mesenchymal cells, which exhibited higher levels of HLA-G expression and lower Notch1 expression (Figs. 2*B* and S2, *C* and *G*). Quantitative image analysis confirmed this observation; single cells expressed higher levels of HLA-G and lower levels of Notch1 than cells in colonies (Figs. 2, *E* and *F* and S2, *E* and *F*). Taken together, EVTs formed on day 6 using a one-step protocol are a heterogeneous mixture of EVTs, reminiscent of heterogeneity *in vivo* between proximal and distal cell columns, and single-cell EVTs. Importantly, because the only difference between the STB and EVT differentiation protocol is the addition of laminin-111 added at passage, we can conclude that laminin-111 switches the terminal trophoblast differentiation fate from STB to EVT during differentiation *in vitro*.

### Assessment of temporal changes in EVT differentiation and the role of ECM proteins

**Figure 3:**
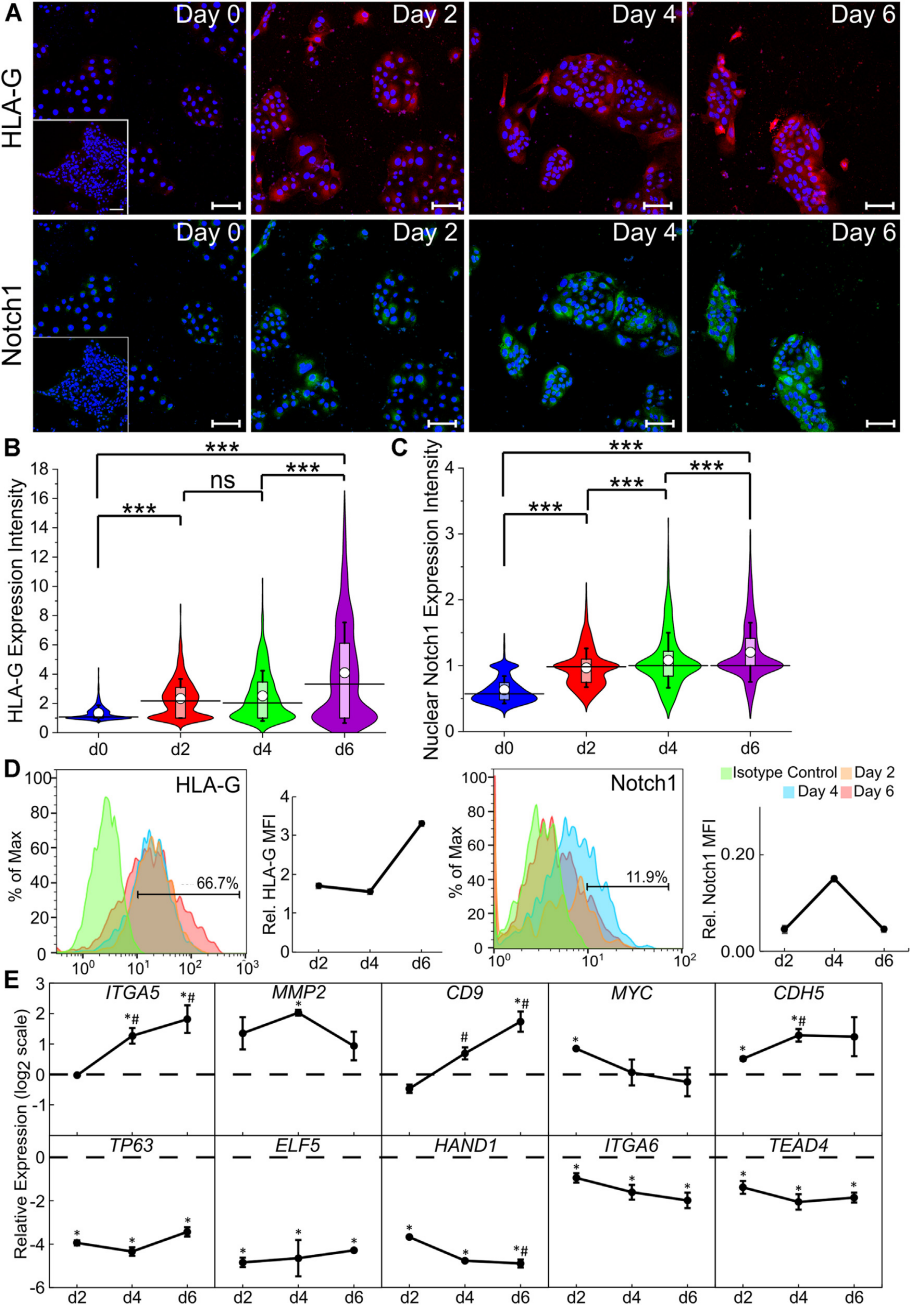
**Assessment of temporal changes in EVT differentiation.***A*, confocal images of CT30 hTSCs on day 0, day 2, day 4, and day 6 of EVT differentiation, staining for HLA-G and Notch1. Nuclei were stained with DAPI. Inset is the respective isotype control. *B*, quantitative analysis of HLA-G expression intensity of CT30 hTSCs on day 0 (n = 1805), day 2 (n = 2179), day 4 (n = 897), and day 6 (n = 1309) of EVT differentiation. Analysis was performed in MATLAB and two biological replicates were used. The *white circle* represents the mean and the *black bar* represents the median (ns, not significant, ∗∗∗*p* < 0.0005). *C*, quantitative analysis of Notch1 expression intensity of CT30 on day 0 (n = 1805), day 2 (n = 2179), day 4 (n = 897), and day 6 (n = 1728) of EVT differentiation. Analysis was performed in MATLAB and two biological replicates were used. The *white circle* represents the mean and the *black bar* represents the median (∗∗∗*p* < 0.0005). *D*, flow cytometry histogram of HLA-G and Notch1 expression of CT30 hTSCs on day 2, day 4, and day 6 of EVT differentiation compared to an isotype control and their relative mean fluorescence intensity (MFI). *E*, gene expression of *ITGA5*, *MMP2*, *CD9*, *MYC*, *CDH5*, *TP63*, *ELF5*, *HAND1*, *ITGA6*, and *TEAD4* of CT30 hTSCs on day 2, day 4, and day 6 of EVT differentiation compared to undifferentiated hTSCs (*dashed line*). Three biological replicates were used (Error bars, S.E., ∗*p* < 0.05 for comparison with undifferentiated hTSCs, ^#^*p* < 0.05 for comparison with cells at day 2, ^%^*p* < 0.05 for comparison with cells at day 4). The scale bars represent 100 μm for all images. EVT, extravillous trophoblast; hTSC, human trophoblast stem cell.

A differentiation protocol without a passage step enabled us to investigate changes in EVT marker expression over a 6-day period. We observed that HLA-G expression increased significantly from day 0 to day 2 and further from day 4 to day 6 (Figs. 3, *A* and *B* and S3, *A* and *B*). Similarly, cells expressed significantly higher levels of Notch1 on day 2 compared with day 0 hTSCs and decreased from day 4 to day 6 of differentiation (Figs. 3, *A* and *C* and S3, *A* and *C*). Consistent with our image analysis, flow cytometry showed that HLA-G expression increased from day 2 to day 6, whereas Notch1 expression increased from day 2 to day 4 but then decreased on day 6 (Figs. 3*D* and S4, *A* and *B*). Together, this is consistent with previous studies that have reported Notch1 expression in early stage EVTs that constitute the proximal column trophoblast and higher HLA-G expression in late stage mesenchymal EVTs (3, 11, 57, 58, 59, 62). Additional qPCR analysis showed increased expression of other EVT markers throughout EVT differentiation. Specifically, in CT30 hTSCs, *ITGA5*, *MMP2*, and *CDH5* expression increased by day 4 of differentiation and *CD9* expression increased by day 6 of differentiation compared with undifferentiated hTSCs at day 0 (Fig. 3*E*). In CT29 hTSCs, *ITGA5*, *ITGA1*, *CD9*, and *CDH5* expression increased by day 6 of differentiation compared with day 0 (Fig. S4*C*). *MYC* expression is associated with the EVT cell columns (3, 59) and was initially upregulated on day 2 in CT30 hTSCs but then decreased back to levels comparable to day 0 hTSCs by day 6 (Fig. 3*E*). Similarly, in CT29 hTSCs, *MYC* expression was significantly reduced on day 6 of EVT differentiation compared with day 0 hTSCs (Fig. S4*C*). Collectively, these data show an increase in mature EVT-associated gene expression over the 6-days differentiation period. Concomitantly, as in the case of STB differentiation, expression of CTB markers decreased during the 6-day differentiation. In CT30 hTSCs *TP63*, *ELF5*, *HAND1*, *ITGA6*, and *TEAD4* expression decreased by day 2 of differentiation (Fig. 3*E*). In CT29 hTSCs, *TP63*, *ELF5*, *HAND1*, *CDH1*, and *TEAD4* were all downregulated by day 6 of EVT differentiation (Fig. S4*C*). The comprehensive dataset from qPCR analysis is included in Tables S1 and S2. Taken together, our results are consistent with a 2-stage EVT differentiation process where hTSCs initially commit to the EVT lineage and gain expression of column EVT markers including Notch1 (day 0–4) and subsequently formation of single-cell, mesenchymal EVTs that express high levels of HLA-G (day 4–6) is observed.

We further investigated whether EVT differentiation is observed in cell culture plates precoated with laminin-111, instead of supplementation in medium as in Figure 2*A*. Briefly, EVT differentiation was carried out on plates coated with a mixture of vitronectin and laminin-111; hTSCs were routinely cultured on plates coated with vitronectin and laminin-521. Use of laminin-111 as a precoating resulted in less efficient differentiation to EVT (Fig. S5, *A* and *D*–*G*). Specifically, HLA-G expression intensity was significantly lower and Notch1 was higher in laminin-111 precoated plates as compared to when laminin-111 is supplemented in the media. Thus, the high concentration of laminin-111 used in the protocol in Figure 2*A* supports efficient EVT differentiation. We also observed some EVT differentiation when collagen IV was used for precoating plates instead of laminin-111. As with laminin-111 precoating, differentiation was not efficient and we observed significantly lower HLA-G and higher Notch1 expression than laminin-111 supplementation (Fig. S5, *B* and *D*–*G*). Nevertheless, it is important to note that there are stark differences in the effect of precoating with laminin-111 and collagen IV *versus* laminin-521 on hTSC differentiation. Use of laminin-521-coated plates during differentiation results in STB differentiation, as discussed in Figure 1. Taken together, these results show that the specific composition of ECM significantly affects hTSC differentiation and high concentrations of laminin-111 (Fig. 2*A*) promotes efficient EVT differentiation. Finally, to investigate whether laminin-111 mediates its effects through integrin-binding, we conducted EVT differentiation as described in Figure 2*A* in the presence of an anti-β1 integrin antibody. Under these conditions, we observed almost complete loss of HLA-G^+^ single cells. Quantitative analysis showed a significant decrease in HLA-G expression and an increase in Notch1 expression (Fig. S5, *C*–*G*). These results show that laminin-111 mediates its effects on EVT differentiation at least in part through interaction with integrins.

### Expression of HIF1α and HIF2α is upregulated during EVT differentiation mediated by laminin-111

**Figure 4:**
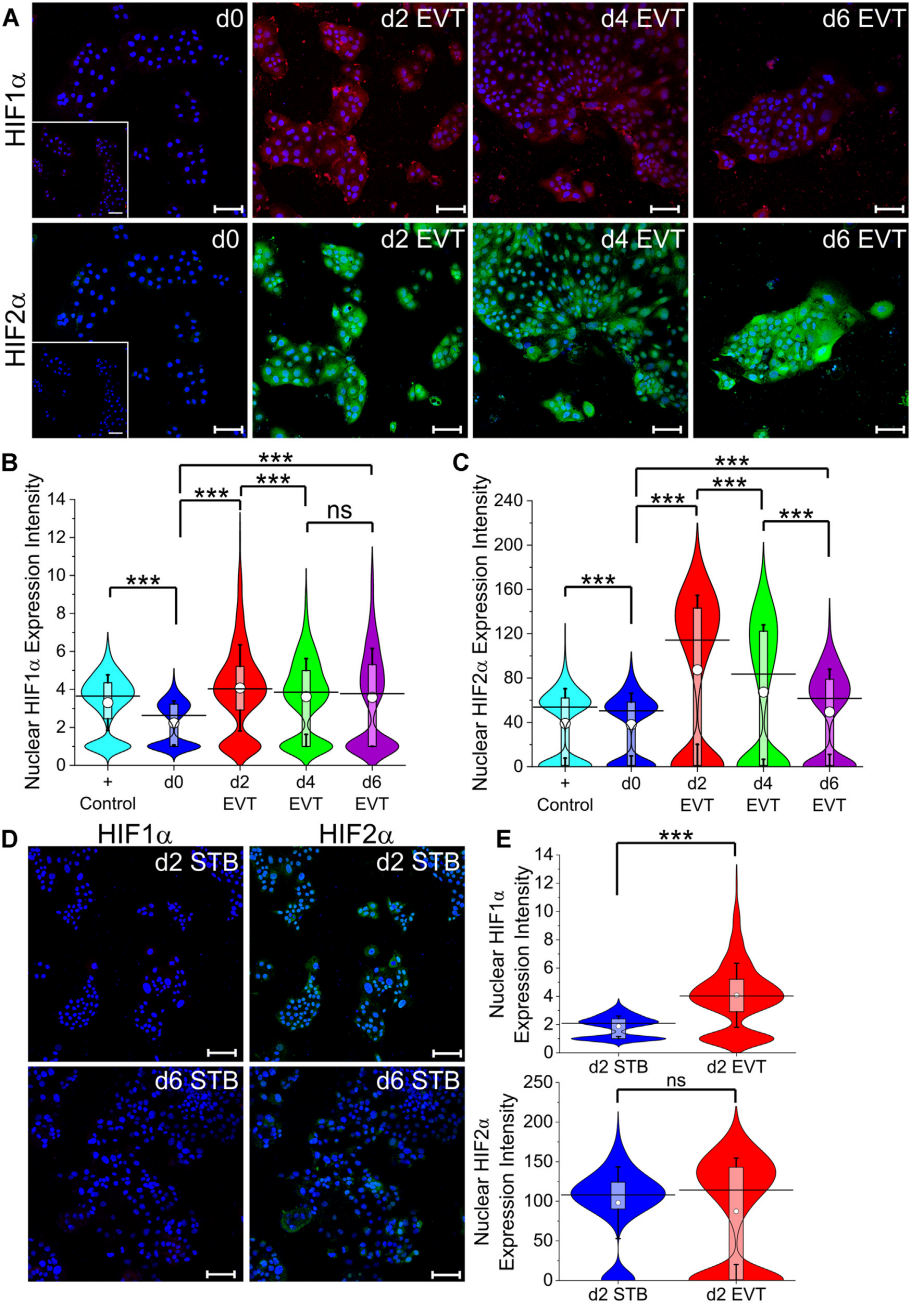
**Expression of HIF1α and HIF2α is upregulated during EVT differentiation mediated by laminin-111.***A*, confocal images of CT30 hTSCs on day 0, day 2, day 4, and day 6 of EVT differentiation, staining for HIF1α and HIF2α. Nuclei were stained with DAPI. Inset is the respective isotype control. *B*, quantitative analysis of HIF1α expression intensity of CT30 hTSCs on day 0 (n = 1298), day 2 (n = 1591), day 4 (n = 1514), and day 6 (n = 2462) of EVT differentiation. Positive control is hTSCs cultured in TSCM with the addition of 10 μM deferoxamine for 2 days. Analysis was performed in MATLAB and two biological replicates were used. The *white circle* represents the mean and the *black bar* represents the median (ns, not significant, ∗∗∗*p* < 0.0005). *C*, quantitative analysis of HIF2α expression intensity of CT30 hTSCs on day 0 (n = 1298), day 2 (n = 1591), day 4 (n = 1514), and day 6 (n = 1640) of EVT differentiation. Positive control is hTSCs cultured in TSCM with the addition of 10 μM deferoxamine for 2 days. Analysis was performed in MATLAB and two biological replicates were used. The *white circle* represents the mean and the *black bar* represents the median (∗∗∗*p* < 0.0005). *D*, confocal images of CT30 hTSCs on day 2 and day 6 of STB differentiation, staining for HIF1α and HIF2α. Nuclei were stained with DAPI. *E*, quantitative analysis of HIF1α and HIF2α expression intensity of CT30 hTSCs on day 2 of STB (n = 638) and EVT (n = 1591) differentiation. Analysis was performed in MATLAB and two biological replicates were used. The *white circle* represents the mean and the *black bar* represents the median (ns, not significant, ∗∗∗*p* < 0.0005). Data for d2 EVT are the same as in panels *B* and *C*. The scale bars represent 100 μm for all images. EVT, extravillous trophoblast; HIF, hypoxia-inducible factor; hTSC, human trophoblast stem cell; TSCM, trophoblast stem cell medium.

Because EVTs invade in low-oxygen tension, before establishment of utero-placental perfusion, the role of hypoxia-inducible factors HIF1α and HIF2α in EVT differentiation has been extensively investigated (72, 73, 74, 75). Notably, HIF1α increases over the course of differentiation from CTB to proximal column EVTs in first-trimester human placenta at both the transcription and protein levels (72). However, over the course of pregnancy, HIF1α expression decreases; highest expression is observed at 5-week gestational age and is almost absent by the 12th week of pregnancy, corresponding to the initiation of utero-placental perfusion and increase in oxygen tension (73). Interestingly, however, in preeclamptic placenta, HIF1α remains upregulated well into mid-gestation (74). Similarly, *EPAS1* (HIF2α) was also found to be upregulated in EVTs from primary tissue compared to CTBs and in EVTs derived from hTSCs described by Okae *et al*. compared to hTSCs at both the transcription and protein levels (75). Importantly, HIF2α expression was more abundant in distal column EVTs (75). Further, specific extracellular matrix cues can direct differentiation of mouse trophoblast stem cells to trophoblast giant cells through a HIF-dependent mechanism, independent of oxygen tension (76). Therefore, we investigated temporal changes in HIF1α and HIF2α expression changed over the course of EVT differentiation in this model. We found that HIF1α and HIF2α was nearly absent in hTSCs but were both significantly expressed during EVT differentiation (Figs. 4*A* and S6*A*), consistent with the literature. We used image analysis to quantify nuclear expression of HIF1α and HIF2α. CT29 hTSCs passaged into TSCM with the addition of 10 μM deferoxamine for 2 days was used as a positive control (Figs. 4, *B* and *C* and S6, *B*, *C* and *F*). Quantitative analysis revealed that both HIF1α and HIF2α expression was increased from hTSCs on day 0 to day 2 of differentiation (Figs. 4, *B* and *C* and S6, *B* and *C*). Interestingly, HIF1α and HIF2α decreased from day 2 to day 4 and day 4 to day 6, respectively, in both CT29 and CT30 hTSCs; nevertheless, HIF1α and HIF2α expression on day 6 remained higher than the expression levels on day 0 (Figs. 4, *B* and *C* and S6, *B* and *C*). Strikingly in contrast, HIF1α and HIF2α expression was barely detected in day 2 or day 6 STB, obtained in the absence of laminin-111 exposure (Figs. 4*D* and S6*D*). Quantitative analysis of day 2 STB and EVT revealed that HIF1α was significantly higher in day 2 EVT than day 2 STB in both CT29 and CT30 hTSCs (Figs. 4, *D* and *E* and S6, *D* and *E*). Collectively, these results show that exposure to laminin-111 results in EVT differentiation and is associated with upregulation of HIF1α and HIF2α.

We further investigated the effect of small molecule inhibitors of FAK and YAP/TAZ signaling on EVT differentiation mediated by laminin-111 exposure. Previous studies have implicated FAK and YAP/TAZ signaling to be downstream of integrin activation in trophoblast (77, 78). Addition of even low concentrations of the FAK inhibitor Y397 (2× IC_50_) resulted in extensive cell death during hTSC differentiation; therefore, no further analysis was possible. Although, high concentrations of the YAP/TAZ inhibitor verteporfin also caused cell death (5× and 10× IC_50_), we could evaluate the effect of verteporfin on EVT differentiation using low concentrations (2× IC_50_). The presence of verteporfin during laminin-111-mediated EVT differentiation did not affect the upregulation of HIFα expression (Fig. S7, *A* and *B*). On the contrary, there was a slight increase in HIF1α expression in both CT29 and CT30 hTSCs. There was a modest but statistically significant increase in Notch1 expression in both CT29 and CT30 hTSCs. HLA-G expression was not altered consistently across both CT29 and CT30 hTSC lines, although HLA-G expression decreased significantly in CT30 hTSCs (Fig. S7, *C* and *D*). Taken together, these results show that inhibition of YAP/TAZ signaling by verteporfin does not prevent upregulation of HIFα or commitment to the EVT lineage upon laminin-111 exposure. Thus, initiation of EVT differentiation by laminin-111 and initial HIFα upregulation is likely independent of YAP/TAZ signaling. However, we cannot rule out the possibility that verteporfin may affect subsequent EVT differentiation, including formation or survival/proliferation of HLA-G^+^ EVTs.

### A defined system enables investigation of TGFβ signaling in EVT differentiation

**Figure 5:**
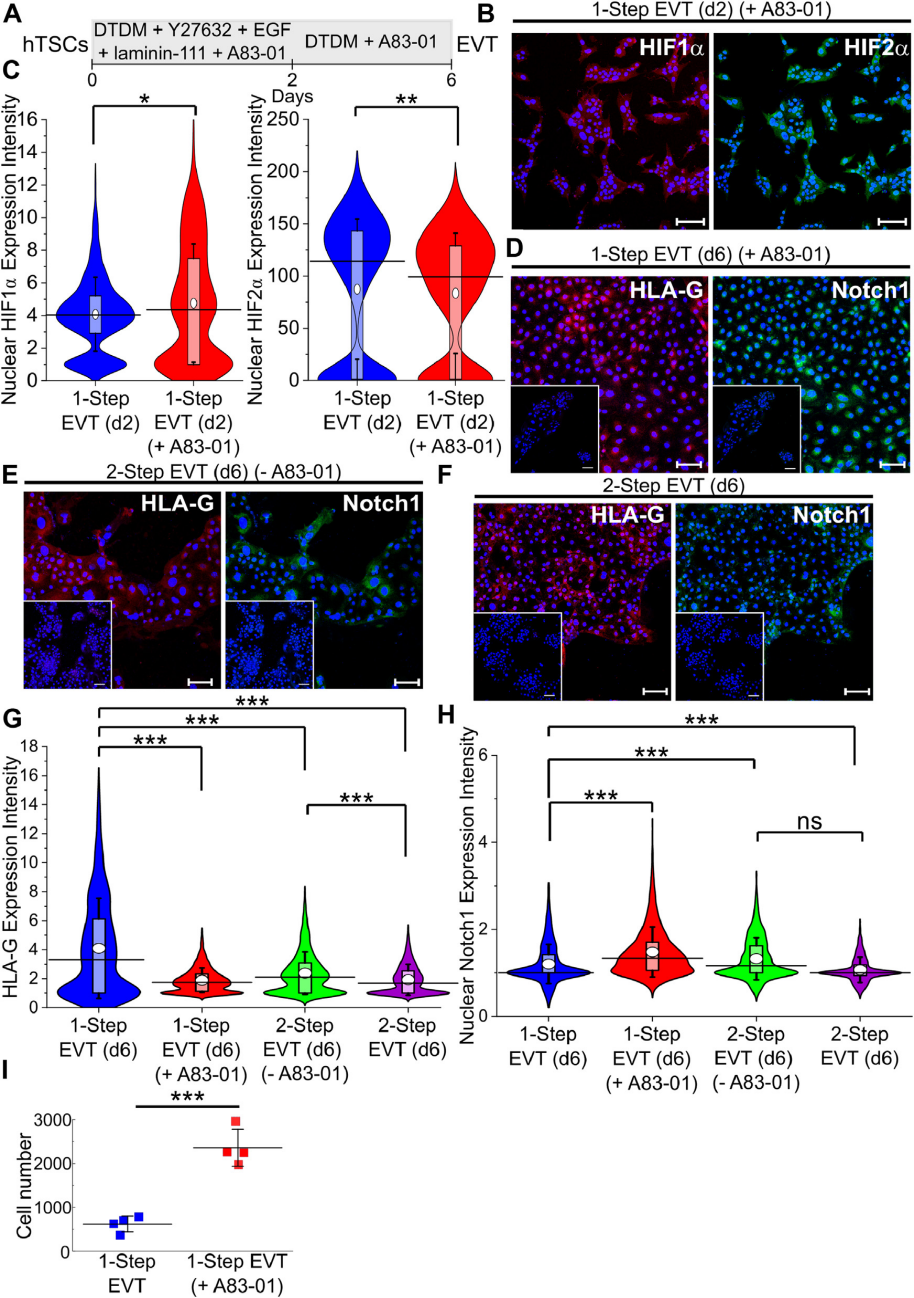
**A defined system enables investigation of TGFβ signaling in EVT differentiation.***A*, schematic of protocol for hTSC differentiation to EVT using the one-step method described in Figure 2*A*, in the presence of the TGFβ inhibitor A83-01. *B*, confocal images of CT30 hTSCs on day 2 of EVT differentiation using the method described in Figure 2*A* (labeled one-step) in the presence of the TGFβ inhibitor, A83-01, staining for HIF1α and HIF2α. Nuclei were stained with DAPI. *C*, quantitative analysis of HIF1α and HIF2α expression intensity of CT30 hTSCs on day 2 of EVT differentiation using the method described in Figure 2*A* in the absence (one-step, n = 1591) or presence of the TGFβ inhibitor, A83-01 (n = 627). Analysis was performed in MATLAB and two biological replicates were used. The *white circle* represents the mean and the *black bar* represents the median (∗*p* < 0.05, ∗∗*p* < 0.005). Data for d2 EVT is the same as used in Figure 4, and Fig. S7. *D*, confocal image of CT30 hTSCs on day 6 of EVT differentiation using the method described in Figure 2*A* (one-step) in the presence of the TGFβ inhibitor, A83-01, staining for HLA-G and Notch1. Nuclei were stained with DAPI. *E*, confocal image of CT30 hTSCs on day 6 of EVT differentiation using the method as previously described (32) (labeled two-step) in the absence of the TGFβ inhibitor, A83-01, staining for HLA-G and Notch1. Nuclei were stained with DAPI. *F*, confocal image of CT30 hTSCs on day 6 of EVT differentiation using the method as previously described (32) (two-step) which includes the TGFβ inhibitor, A83-01, staining for HLA-G and Notch1. Nuclei were stained with DAPI. *G*, quantitative analysis of HLA-G expression intensity of CT30 hTSCs on day 6 of EVT differentiation using the method described in Figure 2*A* (one-step) in the absence (n = 1309) or presence of the TGFβ inhibitor, A83-01 (n = 4219), and the method as previously described (32) (two-step) in the presence (n = 4950) and absence (n = 3311) of A83-01. Analysis was performed in MATLAB and two biological replicates were used. The *white circle* represents the mean and the *black bar* represents the median (∗∗∗*p* < 0.0005). Data for one-step EVT in the absence of A83-01 is same as used in Figure 3, Figs. S5, and S7. *H*, quantitative analysis of Notch1 expression intensity of CT30 hTSCs on day 6 of EVT differentiation using the method described in Figure 2*A* (one-step) in the absence (n = 1728) or presence of the TGFβ inhibitor, A83-01 (n = 4219), and the method as previously described (32) (one-step) in the presence (n = 4950) and absence (n = 3311) of A83-01. Analysis was performed in MATLAB and two biological replicates were used. The *white circle* represents the mean and the *black bar* represents the median (ns, not significant, ∗∗∗*p* < 0.0005). Data for one-step EVT in the absence of A83-01 is same as used in Figure 3, Figs. S5, and S7. *I*, quantitative analysis of the cell number of CT29 and CT30 hTSCs on day 6 of EVT differentiation using the method described in Figure 2*A* (one-step) in the presence and absence of A83-01 (n = 2 × 2 cell lines = 4). Analysis was performed in MATLAB and two biological replicates were used. The black bar represents the mean (∗∗∗*p* < 0.0005). The scale bars represent 100 μm for all images. EVT, extravillous trophoblast; HIF, hypoxia-inducible factor; hTSC, human trophoblast stem cell; TGF, transforming growth factor.

In contrast to previous studies (32), our defined conditions for EVT differentiation do not use TGFβ inhibition. Therefore, we also investigated the effect of including the TGFβ inhibitor, A83-01, during EVT differentiation (Fig. 5*A*). Addition of A83-01 did not affect the upregulation of HIF1α expression across both cell lines, although HIF2α expression exhibited a statistically significant decrease (Figs. 5, *B* and *C* and S8, *A* and *B*). However, in the presence of A83-01, single, mesenchymal EVTs were rarely observed at day 6 (Figs. 5*D* and S8*C*). Further, differentiated cells expressed lower levels of HLA-G and higher levels of Notch1 than the control condition (no A83-01) (Figs. 5, *G* and *H* and S8, *F* and *G*). Further, we also observed an increase in cell number on day 6 in the presence of A83-01, relative to the control (Fig. 5*I*). These data suggest that A83-01 did not prevent upregulation of HIFα expression or initiation of EVT differentiation; however, formation of HLA-G^+^ mesenchymal EVTs was affected. We further compared EVTs differentiated using the method described in this work to those obtained using the method described by Okae *et al.* (32), where TGFβ signaling is inhibited. Briefly, we differentiated hTSCs using the method described by Okae *et al.* (32) in the presence and absence of A83-01 until the sixth day of differentiation; to better compare differentiation using the two methods, cells were not passaged at day 6. EVTs formed using the method described by Okae *et al.* in the absence of A83-01 expressed higher levels of HLA-G than cells differentiated with A83-01, consistent with results obtained using our approach (Figs. 5*G* and S8*F*). However, with or without A83-01, single mesenchymal EVTs were not observed at day 6, without the use of the passage step (Figs. 5, *E* and *F* and S9, *D* and *E*). Further, EVTs obtained using the defined conditions described in this study had higher HLA-G expression than EVTs at day 6 obtained using the protocol described previously by Okae *et al.* (32) with or without A83-01 (Figs. 5*G* and S8*F*). Taken together, these result show that inhibition of TGFβ signaling does not affect commitment to the EVT lineage or upregulation of HIF1α; however, it does prevent the formation of single, mesenchymal HLA-G^+^ cells. Thus, the one-step differentiation model described here can be used to investigate the role of TGFβ signaling during hTSC differentiation.

### A defined system enables investigation of TGFβ signaling in STB differentiation

**Figure 6:**
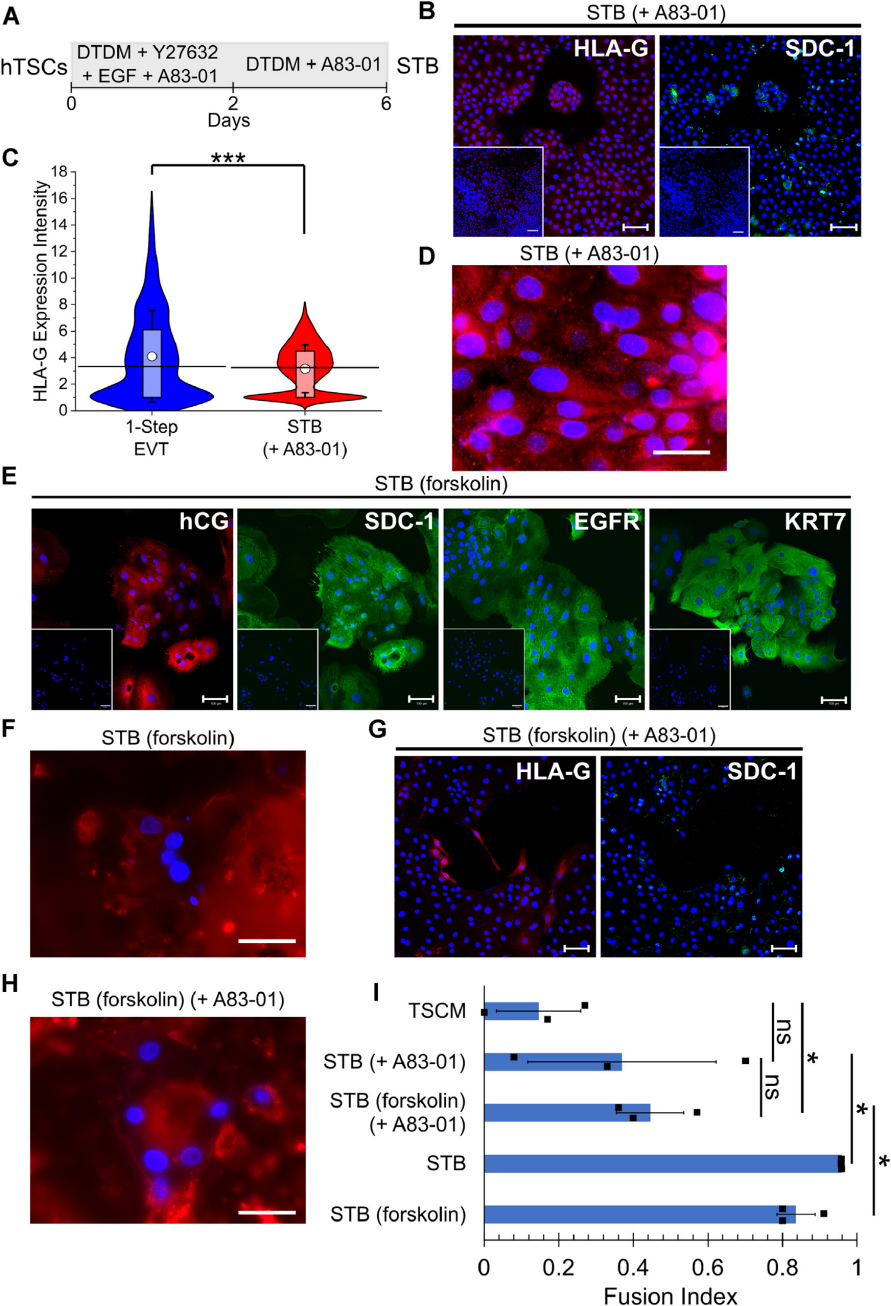
**A defined system enables investigation of TGFβ signaling in STB differentiation.***A*, schematic of protocol for hTSC differentiation to STB in the presence of a TGFβ inhibitor, A83-01. *B*, confocal image of CT30 hTSCs on day 6 of STB differentiation using the method described in Figure 1*A* in the presence of A83-01, staining for HLA-G and SDC-1. Nuclei were stained with DAPI. *C*, quantitative analysis of HLA-G expression intensity of CT30 hTSCs on day 6 of EVT differentiation using the method described in Figure 2*A* (1-Step; n = 1309) or STB differentiation using the method described in Figure 1*A* in the presence of a TGFβ inhibitor, A83-01 (n = 6922). Analysis was performed in MATLAB and two biological replicates were used. The *white circle* represents the mean and the *black bar* represents the median (∗∗∗*p* < 0.0005). Data for one-step EVT is same as used in Figures 3 and 5, Figs. S3, and S7. *D*, fluorescent image of CT30 hTSCs on day 6 of STB differentiation using the method described in Figure 1*A* in the presence of A83-01. Nuclei were stained with DAPI. Membrane was stained with Di-8-ANEPPS cell membrane stain. The scale bar represents 50 μm. *E*, confocal images of CT30 hTSCs on day 6 of STB differentiation using the method using forskolin as previously described (32), staining for hCG, SDC-1, EGFR, and KRT7. Nuclei were stained with DAPI. *F*, fluorescent image of CT30 hTSCs on day 6 of STB differentiation using the method using forskolin as previously described (32). Nuclei were stained with DAPI. Membrane was stained with Di-8-ANEPPS cell membrane stain. The scale bar represents 50 μm. *G*, confocal images of CT30 hTSCs on day 6 of STB differentiation using the method using forskolin as previously described (32) in the presence of the TGFβ inhibitor, A83-01, staining for HLA-G and SDC-1. Nuclei were stained with DAPI. *H*, fluorescent image of CT30 hTSCs on day 6 of STB differentiation using the method using forskolin as previously described (32) in the presence of the TGFβ inhibitor, A83-01. Nuclei were stained with DAPI. Membrane was stained with Di-8-ANEPPS cell membrane stain. The scale bar represents 50 μm. *I*, fusion efficiency of CT30 hTSCs on day 6 of STB differentiation using the method described in Figure 1*A* and the method using forskolin as previously described (32) in the presence and absence of the TGFβ inhibitor, A83-01 compared to CT30 hTSCs cultured in TSCM. Nuclei were stained with DAPI. Membrane was stained with Di-8-ANEPPS cell membrane stain. Three measurements from two biological replicates were used to calculate fusion index. Data for TSCM, STB, and STB (forskolin) is same as used in Figure 1 (ns, not significant, ∗*p* < 0.05, Error bars, SD, n = 3). The scale bars represent 100 μm for all images unless specified otherwise. hTSC, human trophoblast stem cell; STB, syncytiotrophoblast; TGF, transforming growth factor; TSCM, trophoblast stem cell medium.

We had previously found that inhibition of TGFβ signaling in human embryonic stem cells (hESCs)-derived trophoblasts resulted in the formation of EVT, whereas STB was formed in the absence of TGFβ inhibition (40). We therefore also investigated the effect of the TGFβ inhibitor A83-01 on STB differentiation of hTSCs (Fig. 6*A*). Strikingly, we observed that expression of the STB marker SDC-1 was lost in the presence of A83-01; on the other hand, expression of the EVT marker HLA-G expression could be observed (Figs. 6*B* and S9*A*). Indeed, a membrane stain revealed little to no multinucleate syncytia with a fusion index comparable to the negative control (undifferentiated hTSCs) and significantly lower than STB obtained using the protocol described in this work, as shown in Figure 1*A* (Figs. 6, *D* and *I* and S9, *C* and *H*). Thus, inhibition of TGFβ signaling by A83-01 prevented STB differentiation of hTSCs in the absence of forskolin. While we observed HLA-G expression in the presence of A83-01 during STB differentiation, we did not observe single, mesenchymal EVTs that were seen during EVT differentiation initiated by laminin-111 exposure (Figs. 2, *A* and *B* and S2, *B*, *C* and *G*). Quantitative analysis also confirmed that the level of HLA-G expression obtained upon TGFβ inhibition during STB differentiation is significantly lower than that obtained during EVT differentiation as described in Figure 2*A* (Figs. 6*C* and S9*B*). We further investigated the effect of TGFβ inhibition on STB differentiation triggered by exposure to forskolin, as described previously by Okae *et al.* (32). Upon differentiation in the presence of forskolin, we observed multinucleate cells that expressed the STB markers, hCG, SDC-1, EGFR, and KRT7 (Figs. 6, *E* and *F* and S9, *D* and *E*). However, in the presence of A83-01, differentiated cells were no longer multinucleate syncytia, with a fusion index comparable to the hTSC control, consistent with its effects on STB formation using the method described in this work (Figs. 6, *H* and *I* and S9, *G* and *H*). Cells also lost SDC-1 expression and gained HLA-G expression (Figs. 6*G* and S9*F*). Interestingly, in the presence of forskolin, some HLA-G^+^ single mesenchymal EVTs were observed (Figs. 6*G* and S9*F*). Taken together, our results show that TGFβ inhibition prevents STB differentiation of hTSCs.

**Figure 7:**
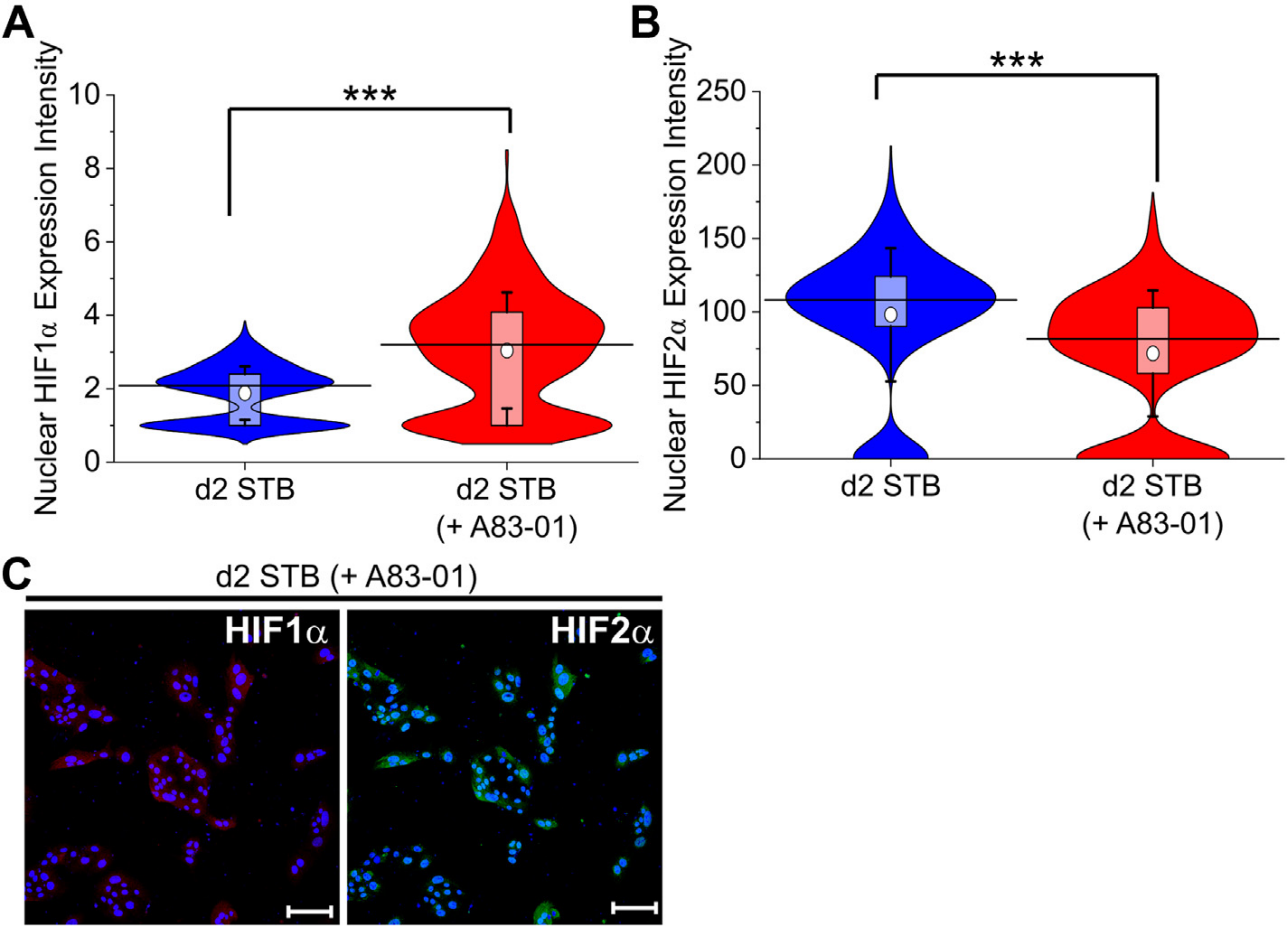
**TGFβ inhibition upregulates HIF1α.***A*, quantitative analysis of HIF1α expression intensity of CT30 hTSCs on day 2 of STB differentiation using the method described in Figure 1*A* in the absence (n = 638) or presence (n = 627) of a TGFβ inhibitor, A83-01. Analysis was performed in MATLAB and two biological replicates were used. The *white circle* represents the mean and the *black bar* represents the median (∗∗∗*p* < 0.0005). Data for d2 STB are the same as used in Figure 4. *B*, Quantitative analysis of HIF2α expression intensity of CT30 hTSCs on day 2 of STB differentiation using the method described in Figure 1*A* in the absence (n = 638) or presence (n = 627) of a TGFβ inhibitor, A83-01. Analysis was performed in MATLAB and two biological replicates were used. The *white circle* represents the mean and the *black bar* represents the median (∗∗∗*p* < 0.0005). Data for d2 STB are the same as used in Figure 4. *C*, confocal images of CT30 hTSCs on day 2 of STB differentiation using the method described in Figure 1*A* in the presence of a A83-01, staining for HIF1α and HIF2α. Nuclei were stained with DAPI. The scale bars represent 100 μm for all images. HIF, hypoxia-inducible factor; hTSC, human trophoblast stem cell; STB, syncytiotrophoblast; TGF, transforming growth factor.

Significantly higher levels of HIFα expression were observed during EVT differentiation in the presence of laminin-111 than STB differentiation the absence of laminin-111 (Fig. 4, *D* and *E* and S6, *D* and *E*). Strikingly, the presence of A83-01 during STB differentiation resulted in a significant increase in HIF1α in both CT29 and CT30 hTSCs on day 2 of differentiation, relative to the control (no A83-01) (Figs. 7, *A* and *C* and S10, *A* and *C*). Interestingly, expression of HIF2α in the presence of A83-01 varied relative to the control in the two cell lines (Figs. 7, *B* and *C* and S10, *B* and *C*). Previous studies have implicated HIFα signaling in inhibition of STB differentiation (72). Therefore, our results suggest that A83-01 may inhibit STB differentiation through upregulation of HIFα. Collectively, our results on the effect of TGFβ inhibition during STB and EVT differentiation show that chemically defined culture conditions can help investigate the role of specific biochemical pathways in trophoblast differentiation.

## Discussion

**Figure 8:**
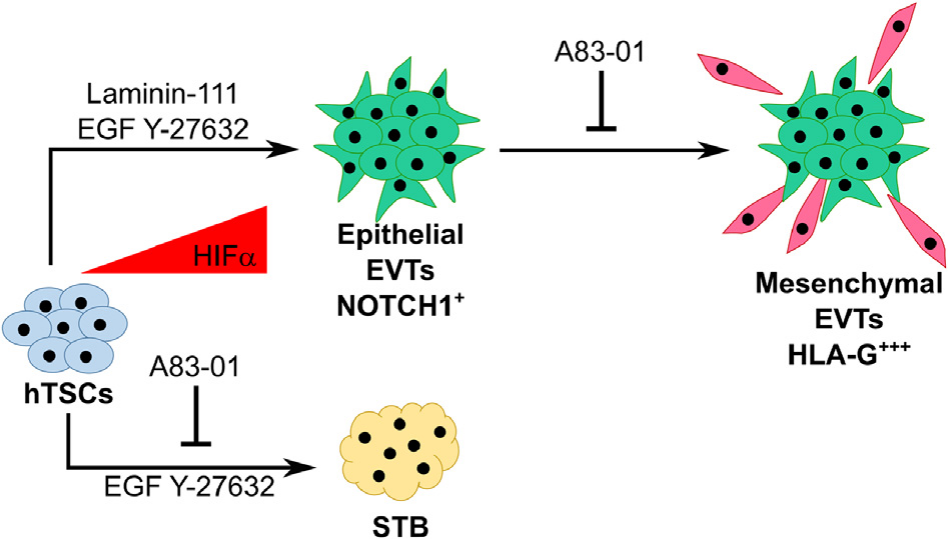
**Laminin-111 switches the terminal trophoblast differentiation fate from STB to EVT.** EVT, extravillous trophoblast; STB, syncytiotrophoblast.

hTSCs derived from the trophectoderm layer of blastocyst-stage embryos, first-trimester placentas, or human pluripotent stem cells can model the CTB cells during early placental development *in vivo*. However, the use of forskolin during STB differentiation of hTSCs or the presence of a TGFβ inhibitor and a passage step during EVT differentiation impede mechanistic studies on hTSC differentiation *in vitro*. Here we present chemically defined conditions for hTSC differentiation to STB and EVT *in vitro*. We show that hTSCs differentiate to STB over a 6-day period, in the absence of forskolin, in a chemically defined medium that is supplemented with EGF and a ROCK inhibitor. Strikingly, short-term (2 days) exposure to a single additional factor during early differentiation, laminin-111, switches the terminal differentiation fate of hTSCs to the EVT lineage (Fig. 8). Notably, differentiation to EVT under these conditions does not involve TGFβ inhibition or an intermediate passage step.

### Cues from extracellular matrix

Exposure to laminin-111 acts as a critical extracellular cue to direct hTSC differentiation to the EVT lineage *in vitro*. A high concentration of laminin-111 is used in our protocol, resulting in the formation of thin layer of substrate overlaying the cells in culture (Fig. S2*A*). Our results are consistent with previous studies by Okae *et al.* (32), where soluble Matrigel was added to cell culture during differentiation in a two-step protocol. Further, our results are also consistent with previous studies on EVT differentiation of trophoblast derived from hESCs (40). hESC-derived trophoblast underwent differentiation to STB, but not EVT, in the absence of TGFβ inhibition in 2D culture; however, EVT differentiation was obtained in a 3D culture with Matrigel with the same culture medium. Taken together with these previous studies, our results implicate a possible role for cues from the extracellular matrix during EVT differentiation. Previous studies that report loss of invasion in primary first-trimester CTBs upon treatment with antibodies against laminin-111 also underscore the role of signals from laminin-111 in EVT differentiation (79). Interestingly, a previous study identified laminin-111 and laminin-511 as key integrin ligands in the mouse trophoblast stem cell niche *in vivo* at E5.5 and ablation of integrin-binding through mutation in the laminin γ1 subunit decreased trophoblast stem cell number *in vivo* (80). In that study, recombinant laminin-111 promoted adhesion of mouse trophoblast stem cells in culture; however, the effect of laminin-111 on trophoblast stem cell differentiation was not investigated. In this context, it is interesting to note that EVT differentiation occurred, albeit less efficiently on cell culture plates coated with a mixture of laminin-111 and vitronectin (Fig. S5, *A* and *D*–*G*). However, STB differentiation is observed under identical conditions on plates coated with mixture of laminin-521 and vitronectin (Figs. 1 and S1). Additionally, hTSCs were cultured on plates coated with a mixture of vitronectin and laminin-521. Thus, specific laminin subunits may provide distinct cues to control hTSC differentiation.

### A single-step protocol captures heterogeneity of cell types and illustrates the multistep process of hTSC differentiation to EVTs

A notable feature of our EVT differentiation protocol is the absence of a passage step that has been previously used to form mesenchymal HLA-G^+^ EVTs (32, 47). The absence of a passage step enables the use of quantitative image analysis to capture of the heterogeneity of cell types that arise as hTSCs differentiate to mature mesenchymal EVTs. Our results show that HIF1α and HIF2α exhibit distinct temporal expression profiles during laminin-111-mediated EVT differentiation. Similarly, expression of Notch1 is upregulated early during EVT differentiation but downregulated in HLA-G^+^ mesenchymal EVTs. These results are consistent with observations *in vivo*, where Notch1 expression is higher in proximal column trophoblasts and decreases in the distal column where EMT occurs (3, 11, 57, 58, 59, 62). Overall, our results suggest that hTSC differentiation *in vitro* can be considered as a two-step process with an initial increase in Notch1 expression in epithelial-like cells, followed by downregulation of Notch1 and increased HLA-G expression in mature mesenchymal cells (Fig. 8).

### Role of HIFα signaling

Despite all our studies being conducted at atmospheric (20%) oxygen, we observe that protein expression and nuclear localization of HIF1α and HIF2α are upregulated during EVT differentiation mediated by laminin-111-exposure. On the other hand, STB differentiation in the absence of laminin-111 does not result in similar upregulation of HIFα. These results are consistent with studies in mouse trophoblast stem cells where specific extracellular matrix cues can direct differentiation to trophoblast giant cells through a HIF-dependent mechanism, independent of oxygen tension (76). Upregulation of HIFα during laminin-111-mediated EVT differentiation occurs even in the presence of the YAP/TAZ inhibitor verteporfin or the TGFβ inhibitor A83-01; this suggests that laminin-111-mediated upregulation of HIFα does not depend on YAP/TAZ or TGFβ signaling. On the other hand, inhibition of TGFβ signaling by A83-01 during STB differentiation (in the absence of laminin-111) resulted in upregulation HIF1α, concomitant with upregulation of the EVT marker HLA-G and abrogation of STB formation. Overall, these results are consistent with a model wherein HIFα signaling mediates initiation of EVT differentiation and inhibition of STB formation, as previously suggested (72).

### Role of TGFβ signaling

Protocols for EVT differentiation of hTSCs have included a TGFβ inhibitor (32, 44, 45, 46, 47). Presence of TGFβ inhibition hinders mechanistic studies on the role TGFβ signaling or extracellular cues that may affect TGFβ signaling, in EVT differentiation. Indeed, our results show that inhibition of TGFβ signaling affects both EVT and STB differentiation of hTSCs. TGFβ inhibition during EVT differentiation in the absence of a passage step resulted in decreased HLA-G expression and single-cell HLA-G^+^ EVTs were rarely observed, suggesting that TGFβ inhibition hinders complete differentiation of hTSCs. These results are consistent with the study by Haider *et al.* (54), where they show that TGFβ signaling is necessary for complete EVT differentiation in a trophoblast organoid model. Our results are also consistent with previous studies wherein E-cadherin+ HTR-8/SVneo cells treated with A83-01 retained a dominant epithelial-like morphology compared to untreated E-Cadherin+ cells, which underwent EMT (81). Further, we observed a loss of STB formation in the presence of TGFβ inhibition, both in the presence or absence of forskolin, suggesting that TGFβ signaling may be necessary for STB formation. These results are consistent with our previous study wherein we reported that activin/nodal/TGFβ signaling switches the terminal differentiation fate of human embryonic stem cell-derived trophoblasts (40). Specifically in that study, inhibition of TGFβ signaling resulted in formation of EVTs; lack of TGFβ inhibition was necessary for STB formation.

In conclusion, we have described a chemically defined culture system for hTSC differentiation *in vitro* that overcome limitations of current approaches. Our results provide baseline conditions for future mechanistic studies on hTSC differentiation.

## Experimental procedures

### Key resources

Key resources for this study are listed in Table 1.

**Table 1:** Key resources

Reagent or resource	Source	Identifier
hTSC cell lines		
CT30 hTSCs	Okae *et al.* (32)	RRID:CVCL_A7BA
CT29 hTSCs	Okae *et al.* (32)	RRID:CVCL_A7BB
SC102A-1 hTSCs	Converted from SC102A-1 hiPSCs from Systems Biosciences (38)	RRID:CVCL_IT66
Antibodies		
Anti-KRT7	Cell Signaling Technologies	Cat#4465, RRID:AB_11178382
Anti-hCG	Abcam	Cat#ab9582, RRID:AB_296507
Anti-P63	Cell Signaling Technologies	Cat#13109, RRID:AB_2637091
Anti-TEAD4	Abcam	Cat#ab58310, RRID:AB_945789
Anti-VE-cadherin	Cell Signaling Technologies	Cat#2500, RRID:AB_10839118
Anti-Notch1	Cell Signaling Technologies	Cat#4380, RRID:AB_10691684
Anti-CD9	Thermo Fisher Scientific	Cat#AHS0902, RRID:AB_1488896
Anti-ErbB2	Cell Signaling Technologies	Cat#2165, RRID:AB_10692490
Anti-EGFR	Cell Signaling Technologies	Cat#4267, RRID:AB 2246311
Anti-HLA-G	Abcam	Cat#ab52455, RRID:AB_880552
Anti-SDC1	Abcam	Cat#ab128936, RRID:AB_11150990
Anti-HIF1α	BD Biosciences	Cat#610959, RRID:AB 398272
Anti-HIF2α	Thermo Fisher Scientific	NB100-122, RRID:AB_10002593
Anti-β1 integrin	Abcam	Cat#ab24693, RRID:AB_448230
PE-conjugated anti-HLA-G	Thermo Fisher Scientific	Cat#12-9957-42, RRID:AB_11149313
PE-conjugated mouse IgG2a	Thermo Fisher Scientific	Cat#IC003P, RRID:AB_357245
Rabbit monoclonal IgG	Abcam	Cat#ab172730, RRID:AB_2687931
Rabbit XP IgG	Cell Signaling Technologies	Cat#3900, RRID:AB_1550038
Rabbit polyclonal IgG	Abcam	Cat#ab37415, RRID:AB 2631996
Mouse IgG1	Abcam	Cat#ab18447, RRID:AB_2722536
Mouse IgG2a	Abcam	Cat#ab18413, RRID:AB_2631983
Mouse IgG2b	Sigma	Cat#MABC006, RRID: AB_97848
Alexa Fluor Plus 488-conjugated anti-rabbit IgG	Thermo Fisher Scientific	Cat#A32731, RRID:AB_2633280
Alexa Fluor Plus 647-conjugated anti-rabbit IgG	Thermo Fisher Scientific	Cat#A32728, RRID:AB_2633277
DAPI	R&D Systems	Cat#5748
Chemicals, Peptides, and Recombinant Proteins		
TrypLE Express	Thermo Fisher Scientific	Cat#12604013
Vitronectin	Thermo Fisher Scientific	Cat#A14700
Laminin-521	Stem Cell Technologies	Cat#77003
SB431542	Tocris	Cat#1614
CYM5541	Tocris	Cat#4897
Y-27632 dihydrochloride	Tocris	Cat#1254
CHIR99021	Tocris	Cat#4423
EGF	Stem Cell Technologies	Cat#78006.2
Y397	Tocris	Cat#3414
Verteporfin	Tocris	Cat#5305
35 mm polystyrene, TC-treated Petri dish	Sarstedt	Cat#83.3900
Cell view glass plates	Greiner Bio-one	Cat#627965
24 well No. 1.5 Coverslip 13 mm Glass Diameter	MatTek	Cat#P24G-1.5-13-F
4% paraformaldehyde in PBS	Thermo Fisher Scientific	Cat#R37814
Triton X-100	Sigma	Cat#T8787
PBS w/o Ca/Mg	Sigma	Cat#D5773
PBS w/Ca/Mg	Sigma	Cat#D8662
Human IgG	Immunoreagents	Cat#Hu-003-C
BSA	Thermo Fisher Scientific	Cat#BP9703
10% BSA fatty acid free in PBS	Sigma	Cat#A1595
VPA	Sigma	Cat#P6273
A83-01	Tocris	Cat#2939
2-mercaptoethanol	Sigma	Cat#M3148
Fetal bovine serum	Thermo Fisher Scientific	Cat#16141-061
DMEM/F12	Thermo Fisher Scientific	Cat#11320033
ITS-X	PeproTech	Cat#00-101
L-ascorbic acid	Sigma	Cat#A8960
Pen/Strep	Thermo Fisher Scientific	Cat#15140122
Laminin-111	R&D Systems	Cat#3446-005-01
Collagen IV	Corning	Cat#354233
Di-8-ANEPPS	Thermo Fisher Scientific	Cat#D3167
Pluronic acid F127	Thermo Fisher Scientific	Cat#P3000MP
RNeasy Mini Kit	Qiagen	Cat#74134
Purelink RNA Mini Kit	Thermo Fisher Scientific	Cat#12183018A
FluoroBrite DMEM	Thermo Fisher Scientific	Cat#A1896701
Software and algorithms		
BioMark HD real-time PCR analysis software	https://www.fluidigm.com/software	N/A
FlowJo v10.7.2	https://www.flowjo.com/solutions/flowjo/downloads	N/A
Fiji	https://imagej.net/software/fiji/downloads	N/A
Zeiss Zen software	https://www.zeiss.com/microscopy/us/products/microscope-software/zen-lite.html	N/A
MATLAB	https://www.mathworks.com/downloads	N/A

Abbreviations: BSA, bovine serum albumin; DMEM, Dulbecco’s Modified Eagle Medium; EGH, epidermal growth factor; HIF, hypoxia-inducible factor; ITS-X, Insulin-Transferrin-Selenium-Ethanolamine.

### hTSC cell culture

hTSCs were cultured as previously described by Okae *et al.* (32) with minor modifications. Cells were cultured in 2 ml of TSCM medium [Dulbecco’s Modified Eagle Medium/Nutrient Mixture F-12 (DMEM/F-12) supplemented with 0.1 mM 2-mercaptoethanol, 0.2% fetal bovine serum, 0.5% Penicillin-Streptomycin (Pen/Strep), 0.3% bovine serum albumin (BSA), 1% Insulin-Transferrin-Selenium-Ethanolamine (ITS-X), 1.5 μg/ml L-ascorbic acid, 50 ng/ml EGF, 2 μM CHIR99021, 0.5 μM A83-01, 1 μM SB431542, 0.8 mM VPA, and 5 μM Y-27632] at 37 °C and 5% CO_2,_ on 35 mm polystyrene plates, precoated with 3 μg/ml of vitronectin and 1 μg/ml of laminin-521. Culture medium was replaced every 2 days. When cells reached confluence, they were dissociated with TrypLE Express for 10 to 15 min at 37 °C and passaged at a 1:10 split ratio. Cells were routinely passaged approximately every 4 to 6 days. All hTSCs used in this study were passaged at least five times prior to use in experiments.

### EVT and STB differentiation

Prior to differentiation, hTSCs at confluence were dissociated into single cells using TrypLE Express and 1.5 × 10^5^ cells were seeded onto a new 35 mm polystyrene or glass plate precoated plate with 3 μg/ml of vitronectin and 1 μg/ml of laminin-521. Where indicated, laminin-111 or collagen IV was replaced with laminin-521 at the same concentration. For STB differentiation, cells were cultured in DTDM [DMEM/F-12 supplemented with 1% ITS-X, 75 μg/ml L-ascorbic acid]. For EVT differentiation, DTDM was supplemented with 150 μg/ml laminin-111 after cells were plated in DTDM. Five μM Y-27632 and 50 ng/ml EGF was added at passage. Cell culture medium was replaced every 2 days and cultures were analyzed at day 6 unless otherwise specified. Two hundred nM verteporfin and 7.5 μM A83-01 were used, where specified. Five μg/ml anti-β1 integrin antibody was added after the addition of laminin, where specified. Two-step EVT and STB differentiation using forskolin were conducted as described, with some minor modifications (32). Briefly, 1.5 × 10^5^ cells were passaged and seeded onto a 35 mm polystyrene or glass plate precoated plate with 3 μg/ml of vitronectin and 1 μg/ml of laminin-521. For EVT differentiation, cells were cultured in EVTM (DMEM/F12 supplemented with 0.1 mM 2-mercaptoethanol, 0.5% Penicillin-Streptomycin, 0.3% BSA, 1% ITS-X supplement, 100 ng/ml NRG1, 7.5 μM A83-01, 2.5 μM Y27632, and 4% KSR). Matrigel was added to a final media concentration of 2% after suspending the cells in EVT medium. On day 3, the medium was replaced with the EVT medium without NRG1 and Matrigel was added to a final concentration of 0.5%. EVTs were fixed on day 6. For STB differentiation, cells were cultured in STBM (DMEM/F12 supplemented with 0.1 mM 2-mercaptoethanol, 0.5% Penicillin-Streptomycin, 0.3% BSA, 1% ITS-X supplement, 2.5 μM Y27632, 2 μM forskolin, and 4% KSR). Media was replaced on day 3 and cells were fixed on day 6.

### Immunostaining

For immunofluorescence analysis, 3 × 10^4^ cells were grown on 24-well glass bottom plates coated with 3 μg/ml of vitronectin and 1 μg/ml of laminin-521. Where indicated, 2× or 4× this number of cells were plated. Cells were fixed with 4% paraformaldehyde fixative solution for 5 min, permeabilized with 0.5% Triton X-100 in PBS for 10 min, then blocked in blocking buffer [0.5% BSA, and 200 μM human IgG in PBS] for at least 1 h. Cells were then incubated overnight at 4 °C in primary antibody diluted in blocking buffer. Primary antibodies used were anti-HLA-G (1:250), anti-VE-cadherin (1:250), anti-Notch1 (1:200), anti-CD9 (1:50), anti-ErbB2 (1:250), anti-EGFR (1:50), anti-hCG (1:50), anti-SDC-1 (1:250), anti-KRT7 (1:50), anti-p63 (1:250), anti-HIF1α (1:100), and HIF2α (1:100). Secondary antibodies were added an hour before imaging. Corresponding isotype controls (rabbit monoclonal IgG, rabbit XP IgG, rabbit polyclonal IgG, mouse IgG1, mouse IgG2a, and mouse IgG2b) were used at primary antibody concentrations. Alexa Fluor 488- or Alexa Fluor 647-conjugated secondary antibodies were used. Nuclei were stained with DAPI and images were taken with a laser scanning confocal microscope (LSM880, Carl Zeiss).

### Confocal image analysis

Image analysis was conducted using an image processing algorithm created in MATLAB R2021a. All image processing was performed *post hoc*. DAPI (blue channel) was isolated from the RGB image, binarized, and processed to accurately represent the number of cells in each image. Two images with known cell number were used to develop the processing steps and these were then extrapolated to all other images. The primary antibody stain of interests (red and green) was isolated and processed in the same manner. The average intensity of the red and green stains nearest each cell was assigned as the average expression intensity for that cell. If the nearest red or green stain was farther than the nearest blue stain, then the cell was assigned the average isotype control expression value. This was performed for 1 to 3 isotype control images and 14 experimental images (seven images for each of the two replicates). For analysis labeled nuclear, only pixels that overlapped-DAPI pixels were used for average expression intensity. For quartile analysis, cells negative for both HLA-G and Notch1 were not included. Data was normalized by the average isotype control expression intensity.

### Statistical analysis

For immunofluorescence analysis, statistical analysis was conducted using the nonparametric Mann-Whitney *U* test because the data is not normally distributed. The analysis was performed in Microsoft Excel using the test for large sample sizes (82). Results of this test are given as a *p*-value to compare differences in medians. For all other statistical analyses, a two-tailed Student’s *t* test with either unequal or equal variance was used, depending on results from a two-sample F-test for equality of variances. Statistical significance was inferred at *p* < 0.05.

### Membrane staining

hTSCs and STB were cultured as described here or by Okae *et al.* (32). Cells were washed and subsequently incubated with 1 to 2 μM Di-8-ANEPPS and DAPI on ice for at least 1 h. Cells were washed once and imaged in FluoroBrite DMEM using a Keyence BZ-X810 system. Nuclei and syncytia were counted manually to determine fusion efficiency.

### Flow cytometry

Cells were dissociated with TrypLE and fixed with 2% paraformaldehyde for 10 min in suspension. They were then blocked in Saponin blocking buffer [1% BSA 1 mg/ml Saponin] for 15 min at room temperature. PE-conjugated anti-HLA-G antibody (1:20) or anti-Notch1 antibody (1:800) diluted in Saponin-blocking buffer was then added and the cells incubated for 1 h on ice. Alexa-Fluor Plus 488-conjugated rabbit IgG was added as a secondary to Notch1 antibody staining and incubated for an additional hour on ice. PE-conjugated mouse IgG2a or Rabbit XP IgG was used as the isotype control. Flow cytometry was carried out using a MACSQuant VYB and the data were analyzed using FlowJo software (Table 1).

### RNA extraction, cDNA synthesis, and quantitative reverse-transcription PCR (RT-qPCR)

RNA was isolated using Invitrogen PureLink RNA Mini Kit (Thermo Fisher Scientific) or the RNeasy Mini Kit (Qiagen) using the manufacturers’ protocol. RNA was quantified using a Nanodrop 1000 spectrophotometer (Thermo Fisher Scientific). One μl of RNA was transformed into complementary DNA using Fluidigm Reverse Transcription Master Mix (Fluidigm) according to the manufacturer’s protocol and underwent 16 preamplification cycles using the Fluidigm Preamp Master Mix according to the manufacturer’s protocol. RNA was then analyzed by the UNC Advanced Analytics Core facility using the Fluidigm Biomark HD 96.96 IFC array (Fluidigm) and validated TaqMan probes according to the manufacturer’s protocol. Using BioMark HD software (Fluidigm, Table 1), Ct values were then normalized against the geometric mean of GAPDH and beta-actin (ACTB), and relative log_2_fold changes were calculated, normalizing to day 0 hTSCs based on the ΔΔCT method.

## Data availability

The code used for image analysis can be found at https://doi.org/10.5281/zenodo.7700524. All other data that support the findings of this study are available within the article and its supplementary methods.

## Supporting information

This article contains supporting information.

## Conflict of interest

The authors declare that they have no conflicts of interest with the contents of this article.
