# Supplementary material for: Laminin switches terminal differentiation fate of human trophoblast stem cells under chemically defined culture conditions
Source: J Biol Chem. 2023 Mar 25;299(5):104650. doi: 10.1016/j.jbc.2023.104650 (PMC10176266; doi:10.1016/j.jbc.2023.104650)
Supplement: Supporting Figures S1–S10 [file mmc2.pdf]

Laminin switches terminal differentiation fate of human trophoblast stem cells under chemically defined culture conditions.

Victoria Karakis[1], Mahe Jabeen[1], John W. Britt[2], Abigail Cordiner[1], Adam Mischler[1], Feng Li[3], Adriana San Miguel[1], Balaji M Rao[1, 4]

[1]Department of Chemical and Biomolecular Engineering, North Carolina State University, Raleigh, North Carolina 27695

[2]Department of Genetics, North Carolina State University, Raleigh, North Carolina 27607

[3]Department of Pathology and Laboratory Medicine, University of North Carolina-Chapel Hill, Chapel Hill, North Carolina 27599

[4]Golden LEAF Biomanufacturing Training and Education Center, North Carolina State University, Raleigh, North Carolina 27695

Corresponding author: Dr. Balaji Rao (bmrao@ncsu.edu)

Supporting Information

Supplementary Figures S1-S10

Supplementary Tables S1, S2 (included as a separate file)

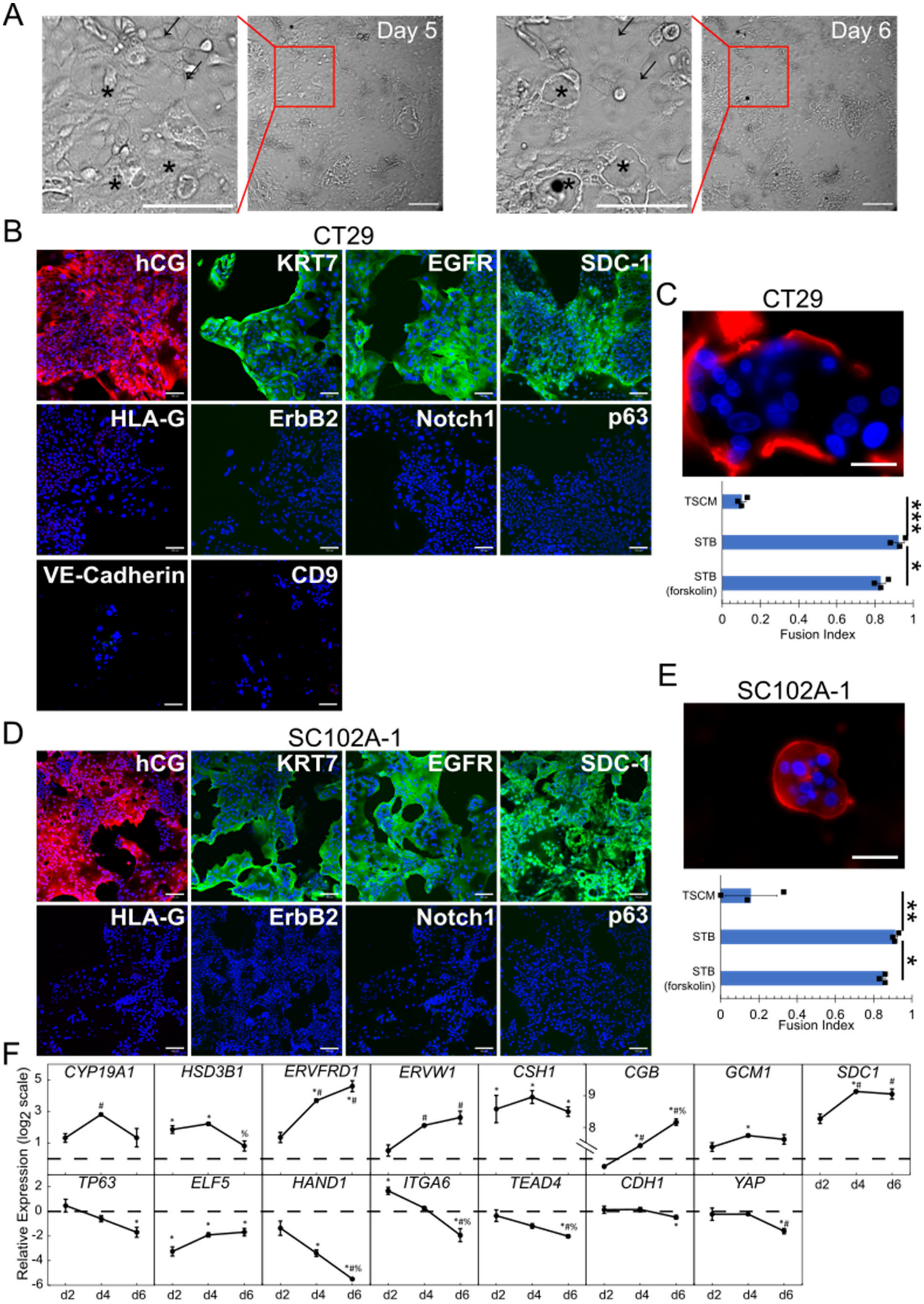
A
Day 5
Day 6
B
CT29
hCG
KRT7
EGFR
SDC-1
HLA-G
ErbB2
Notch1
p63
VE-Cadherin
CD9
C
CT29
TSCM
STB
STB (forskolin)
Fusion Index
D
SC102A-1
E
SC102A-1
F
CYP19A1
HSD3B1
ERVFRD1
ERVW1
CSH1
CGB
GCM1
SDC1
TP63
ELF5
HAND1
ITGA6
TEAD4
CDH1
YAP
Relative Expression (log2 scale)
d2
d4
d6

Figure S1: Chemically defined conditions for STB differentiation in the absence of forskolin.

(A) Bright field images of CT30 hTSCs on day 5 and day 6 of STB differentiation. Outcrop is the magnified image. On day 6, stars indicate where lacunae formed, and arrows indicate where cell boundaries fused. In these same positions on day 5, no lacunae are seen, and distinct cell boundaries are present.

(B) Confocal images of CT29 hTSCs on day 6 of STB differentiation, staining for hCG, KRT7, EGFR, SDC-1, VE-Cadherin, HLA-G, ErbB2, Notch1, p63, and CD9. Nuclei were stained with DAPI.

(C) Fluorescent image and fusion efficiency of CT29 hTSCs on day 6 of STB differentiation using the method described in Fig. 1A and the method using forskolin as previously described (32), compared to CT29 hTSCs cultured in TSCM. Nuclei were stained with DAPI. Membrane was stained with Di-8-ANEPPS cell membrane stain. Three measurements from two biological replicates were used to calculate fusion index.. Scale bar is 50 μm. (*p<0.05, ***p<0.0005, Error bars, S.D., n=3).

(D) Confocal images of SC102A-1 hTSCs on day 6 of STB differentiation, staining for hCG, KRT7, EGFR, SDC-1, HLA-G, ErbB2, Notch1, and p63. Nuclei were stained with DAPI.

(E) Fluorescent image and fusion efficiency of SC102A-1 hTSCs on day 6 of STB differentiation using the method described in Fig. 1A and the method using forskolin as previously described (32), compared to SC102A-1 hTSCs cultured in TSCM. Nuclei were stained with DAPI. Membrane was stained with Di-8-ANEPPS cell membrane stain. Three measurements from two biological replicates were used to calculate fusion index. Scale bar is 50 μm. (*p<0.05, **p<0.005, Error bars, S.D., n=3).

(F) Gene expression of *CYP19A1, HSD3B1, ERVFRD1, ERVW1, CSH1, CGB, GCM1, SDC-1, TP63, ELF5, HAND1, ITGA6, TEAD4, CDH1,* and *YAP* of CT29 hTSCs on day 2, day 4, and day 6 of STB differentiation compared to undifferentiated hTSCs (dashed line). Three biological replicates were used. (Error bars, S.E., *p<0.05 for comparison with undifferentiated hTSCs, $^{\#}$p<0.05 for comparison with cells at day 2, $^{\%}$p<0.05 for comparison with cells at day 4).

Scale bars are 100μm for all images unless specified otherwise.

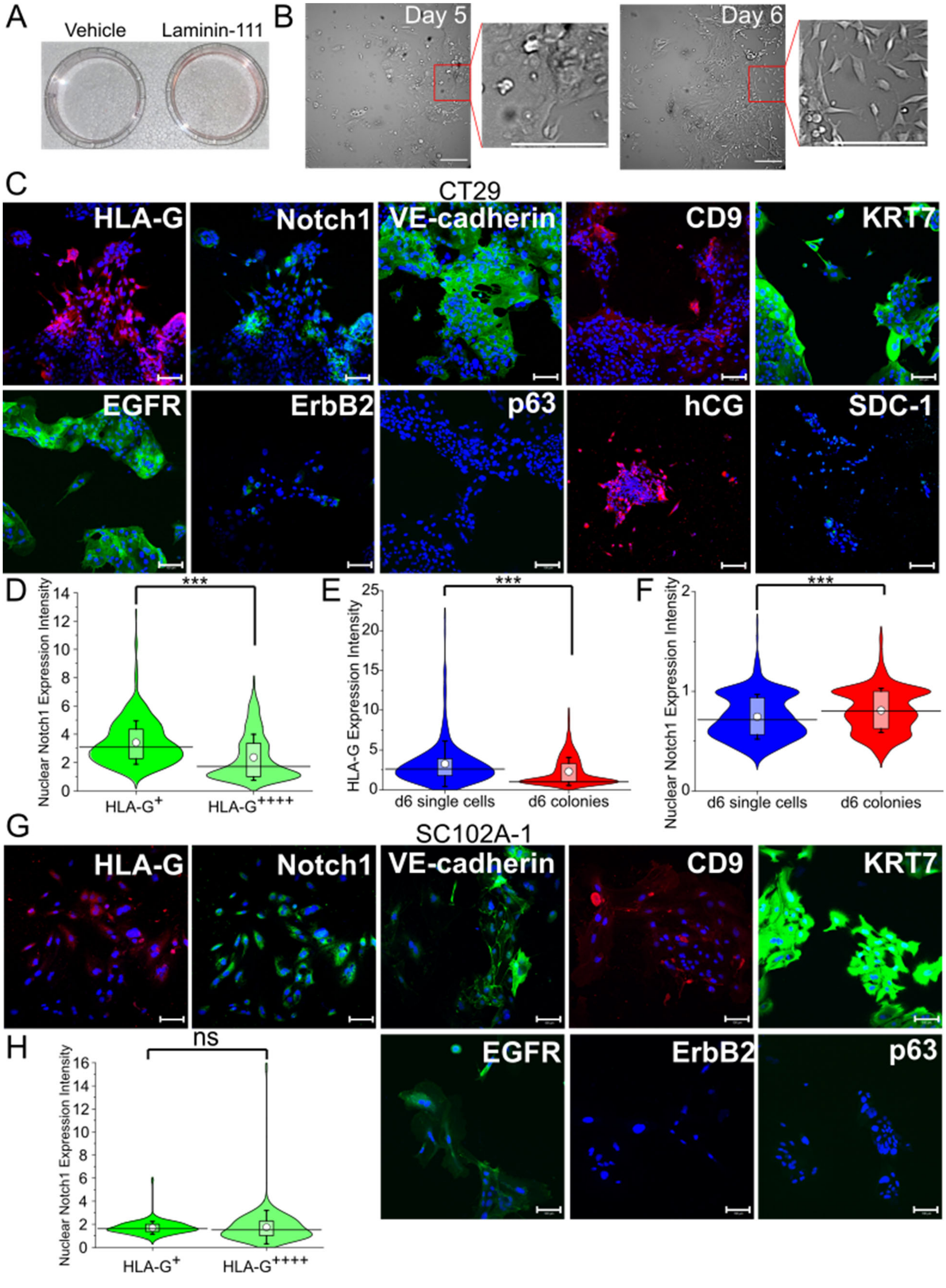
A
Vehicle
Laminin-111
B
Day 5
Day 6
C
CT29
HLA-G
Notch1
VE-cadherin
CD9
KRT7
EGFR
ErbB2
p63
hCG
SDC-1
D
***
Nuclear Notch1 Expression Intensity
HLA-G+
HLA-G++++
E
***
HLA-G Expression Intensity
d6 single cells
d6 colonies
F
***
Nuclear Notch1 Expression Intensity
d6 single cells
d6 colonies
G
SC102A-1
HLA-G
Notch1
VE-cadherin
CD9
KRT7
H
ns
Nuclear Notch1 Expression Intensity
HLA-G+
HLA-G++++
EGFR
ErbB2
p63

Figure S2: Presence of laminin-111 switches hTSC differentiation from STB to EVT fate.

(A) Image of plastic plates with dyed-red DMSO (Vehicle) or dyed-red laminin-111. 75 μL of DMSO or laminin-111 was dyed with red food coloring and was added to 2 mL of DMEM on pre-coated plastic plates. The plates were incubated at 37°C and 5% CO_2 for 2 days after which the liquid media was aspirated. The DMSO is completely removed from the plate after aspiration (Vehicle) whereas dyed-red laminin-111 remains present on the plate (Laminin-111).

(B) Bright field images of CT30 hTSCs on day 5 and day 6 of EVT differentiation. Outcrop is the magnified image. Cells on day 5 are in an epithelial colony but on day 6 single, mesenchymal cells are observed in the same location.

(C) Confocal images of CT29 hTSCs on day 6 of EVT differentiation, staining for HLA-G, Notch1, VE-Cadherin, CD9, KRT7, EGFR, ErbB2, p63, hCG, and SDC-1. Nuclei were stained with DAPI.

(D) Quantitative analysis of Notch1 expression intensity of CT29 hTSCs on day 6 of EVT differentiation from the bottom ($HLA\text{-}G^{+}$) and top ($HLA\text{-}G^{++++}$) 25% of HLA-G expression intensity cells (n=550, each). Analysis was performed in MATLAB and two biological replicates were used. The white circle represents the mean and the black bar represents the median. (***$p<0.0005$).

(E) Quantitative analysis of HLA-G expression intensity of CT29 hTSCs on day 6 of EVT differentiation grouped into two categories: cells with no neighboring cells within a radius of 50 μm (n=211) labeled as d6 single cells or cells with at least 1 or more neighboring cells within a 50 μm radius (n=518) labeled as d6 colonies. Analysis was performed in MATLAB and two biological replicates were used. The white circle represents the mean and the black bar represents the median. (***$p<0.0005$).

(F) Quantitative analysis of Notch1 expression intensity of CT29 hTSCs on day 6 of EVT differentiation grouped into two categories: cells with no neighboring cells within a radius of 50 μm (n=645) labeled as d6 single cells or cells with at least 1 or more neighboring cells within a 50 μm radius (n=770) labeled as d6 colonies. Analysis was performed in MATLAB and two biological replicates were used. The white circle represents the mean and the black bar represents the median. (***$p<0.0005$).

(G) Confocal images of SC102A-1 hTSCs on day 6 of EVT differentiation, staining for HLA-G, Notch1, VE-Cadherin, CD9, KRT7, EGFR, ErbB2, and p63. Nuclei were stained with DAPI.

(H) Quantitative analysis of Notch1 expression intensity of SC102A-1 hTSCs on day 6 of EVT differentiation from the bottom ($HLA\text{-}G^{+}$) and top ($HLA\text{-}G^{++++}$) 25% of HLA-G expression intensity cells (n=135, each). Analysis was performed in MATLAB and two biological replicates were used. The white circle represents the mean and the black bar represents the median. (ns, not significant).

Scale bars are 100μm for all images.

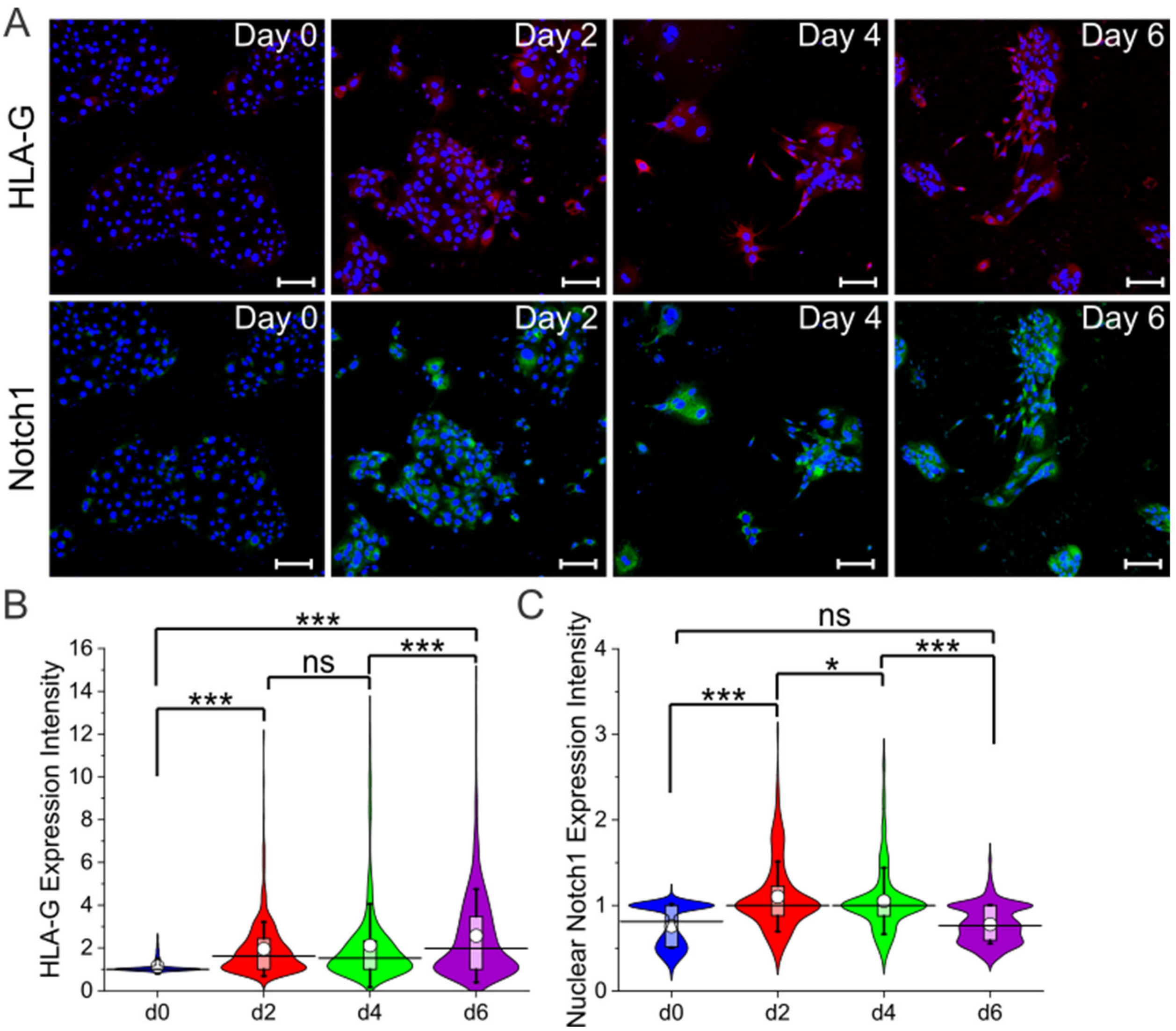

Figure S3: Assessment of temporal changes in EVT differentiation.

(A) Confocal images of CT29 hTSCs on day 0, day 2, day 4, and day 6 of EVT differentiation, staining for HLA-G and Notch1. Nuclei were stained with DAPI.

(B) Quantitative analysis of HLA-G expression intensity of CT29 hTSCs on day 0 (n=2566), day 2 (n=822), day 4 (n=994), and day 6 (n=729) of EVT differentiation. Analysis was performed in MATLAB and two biological replicates were used. The white circle represents the mean and the black bar represents the median. (ns, not significant, ***p<0.0005).

(C) Quantitative analysis of Notch1 expression intensity of CT29 hTSCs on day 0 (n=2566), day 2 (n=822), day 4 (n=994), and day 6 (n=1415) of EVT differentiation. Analysis was performed in MATLAB and two biological replicates were used. The white circle represents the mean and the black bar represents the median. (ns, not significant, *p<0.05, ***p<0.0005).

Scale bars are 100μm for all images.

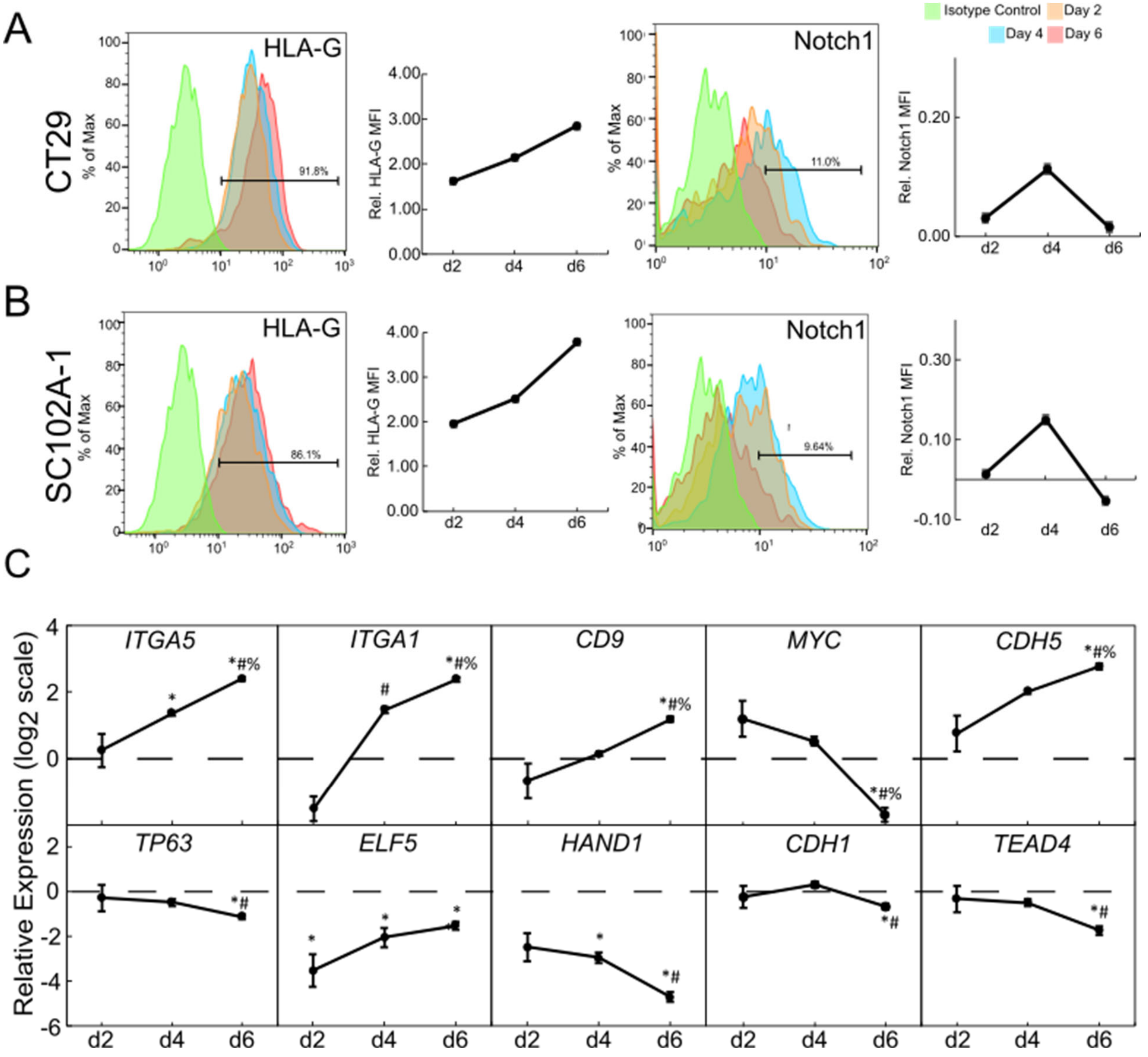

Figure S4: Assessment of temporal changes in EVT differentiation.

(A) Flow cytometry histogram of HLA-G and Notch1 expression of CT29 hTSCs on day 2, day 4, and day 6 of EVT differentiation compared to an isotype control and their relative mean fluorescence intensity (MFI).

(B) Flow cytometry histogram of HLA-G and Notch1 expression of SC102A-1 hTSCs on day 2, day 4, and day 6 of EVT differentiation compared to an isotype control and their relative mean fluorescence intensity (MFI).

(C) Gene expression of *ITGA5, CD9, ITGA1, MYC, CDH5, TP63, ELF5, HAND1, CDH1,* and *TEAD4* of CT29 hTSCs on day 2, day 4, and day 6 of EVT differentiation compared to undifferentiated hTSCs (dashed line). Three biological replicates were used. (Error bars, S.E., *$p<0.05$ for comparison with undifferentiated hTSCs, $^{\#}p<0.05$ for comparison with cells at day 2, $^{\%}p<0.05$ for comparison with cells at day 4).

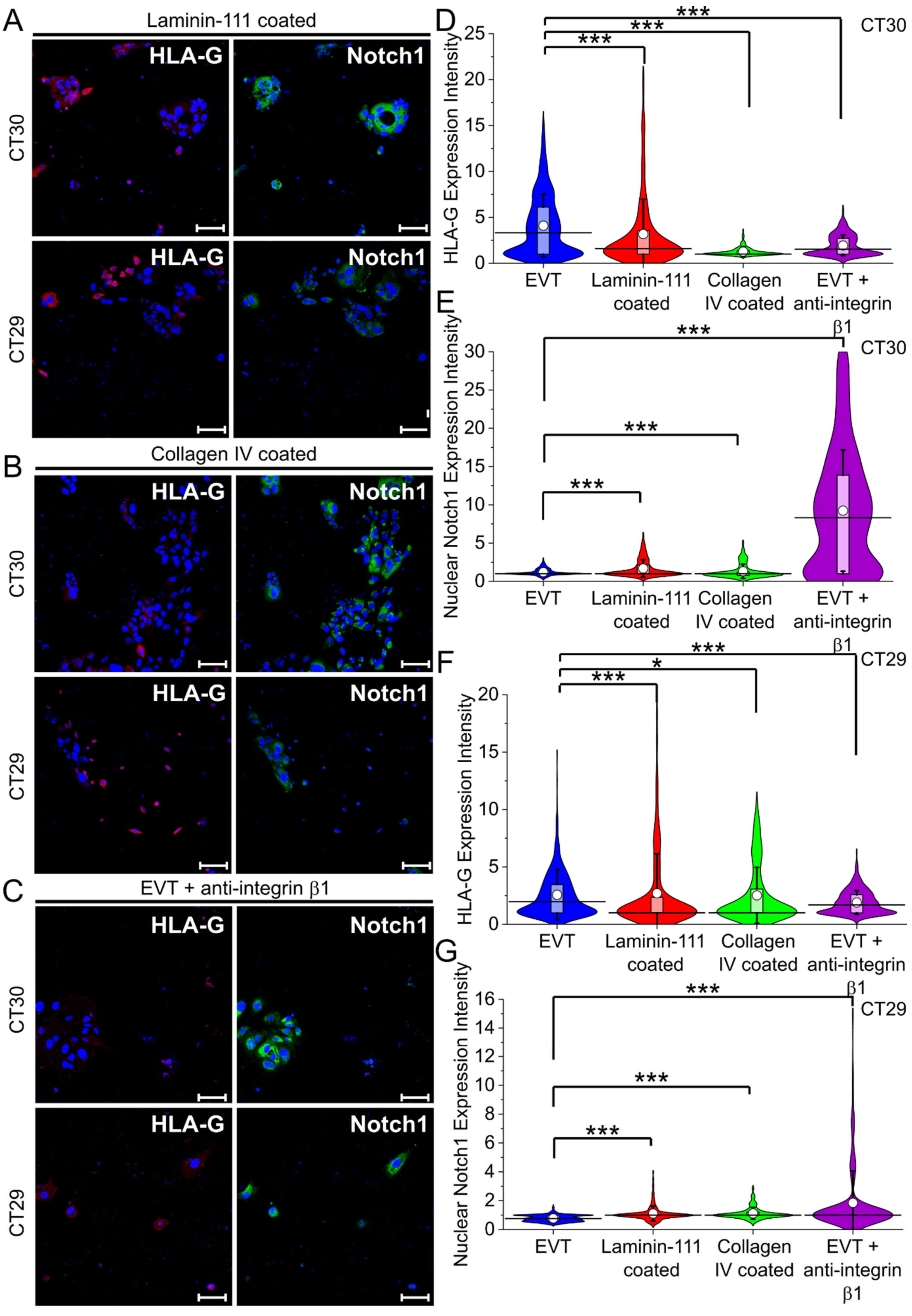
A
Laminin-111 coated
HLA-G
Notch1
CT30
CT29
B
Collagen IV coated
C
EVT + anti-integrin β1
D
CT30
HLA-G Expression Intensity
***
EVT
Laminin-111 coated
Collagen IV coated
EVT + anti-integrin β1
E
Nuclear Notch1 Expression Intensity
F
CT29
*
G

Figure S5: Assessment of the role of ECM proteins.

(A) Confocal images of CT30 and CT29 hTSCs on day 6 of EVT differentiation using exposure to laminin-111-coated plates, staining for HLA-G and Notch1. The same concentration for vitronectin was also used. Nuclei were stained with DAPI.

(B) Confocal images of CT30 and CT29 hTSCs on day 6 of EVT differentiation using exposure to collagen IV-coated plates, staining for HLA-G and Notch1. The same concentration for vitronectin was also used. Nuclei were stained with DAPI.

(C) Confocal images of CT30 and CT29 hTSCs on day 6 of EVT differentiation using the method described in Fig. 2A in the presence of an anti-β1 integrin antibody, staining for HLA-G and Notch1. Nuclei were stained with DAPI.

(D) Quantitative analysis of HLA-G expression intensity of CT30 hTSCs on day 6 of EVT differentiation using exposure to laminin-111-coated plates (n=555), collagen IV-coated plates (n=330), or from the method described in Fig. 2A to laminin-111 described in Fig. 2A with (n=351) and without (n=1309) the addition of an anti-β1 integrin antibody. Analysis was performed in MATLAB and two biological replicates were used. The white circle represents the mean and the black bar represents the median. (***$p<0.0005$). Data for EVT is same as in Fig. 3.

(E) Quantitative analysis of Notch1 expression intensity of CT30 hTSCs on day 6 of EVT differentiation using exposure to laminin-111-coated plates (n=555), collagen IV-coated plates (n=330), or the method described in Fig. 2A with (n=351) and without (n=1728) the addition of an anti-β1 integrin antibody. Analysis was performed in MATLAB and two biological replicates were used. The white circle represents the mean and the black bar represents the median. (***$p<0.0005$). Data for EVT is same as in Fig. 3.

(F) Quantitative analysis of HLA-G expression intensity of CT29 hTSCs on day 6 of EVT differentiation using exposure to laminin-111-coated plates (n=851), collagen IV-coated plates (n=255), or the method described in Fig. 2A with (n=630) and without (n=729) the addition of an anti-β1 integrin antibody. Analysis was performed in MATLAB and two biological replicates were used. The white circle represents the mean and the black bar represents the median. (*$p<0.05$, ***$p<0.0005$). Data for EVT is same as used in Fig. S3.

(G) Quantitative analysis of Notch1 expression intensity of CT29 hTSCs on day 6 of EVT differentiation using exposure to laminin-111-coated plates (n=851), collagen IV-coated plates (n=255), or the method described in Fig. 2A with (n=630) and without (n=1415) the addition of an anti-β1 integrin antibody. Analysis was performed in MATLAB and two biological replicates were used. The white circle represents the mean and the black bar represents the median. (***$p<0.0005$). Data for EVT is same as used in Fig. S3.

Scale bars are 100µm for all images.

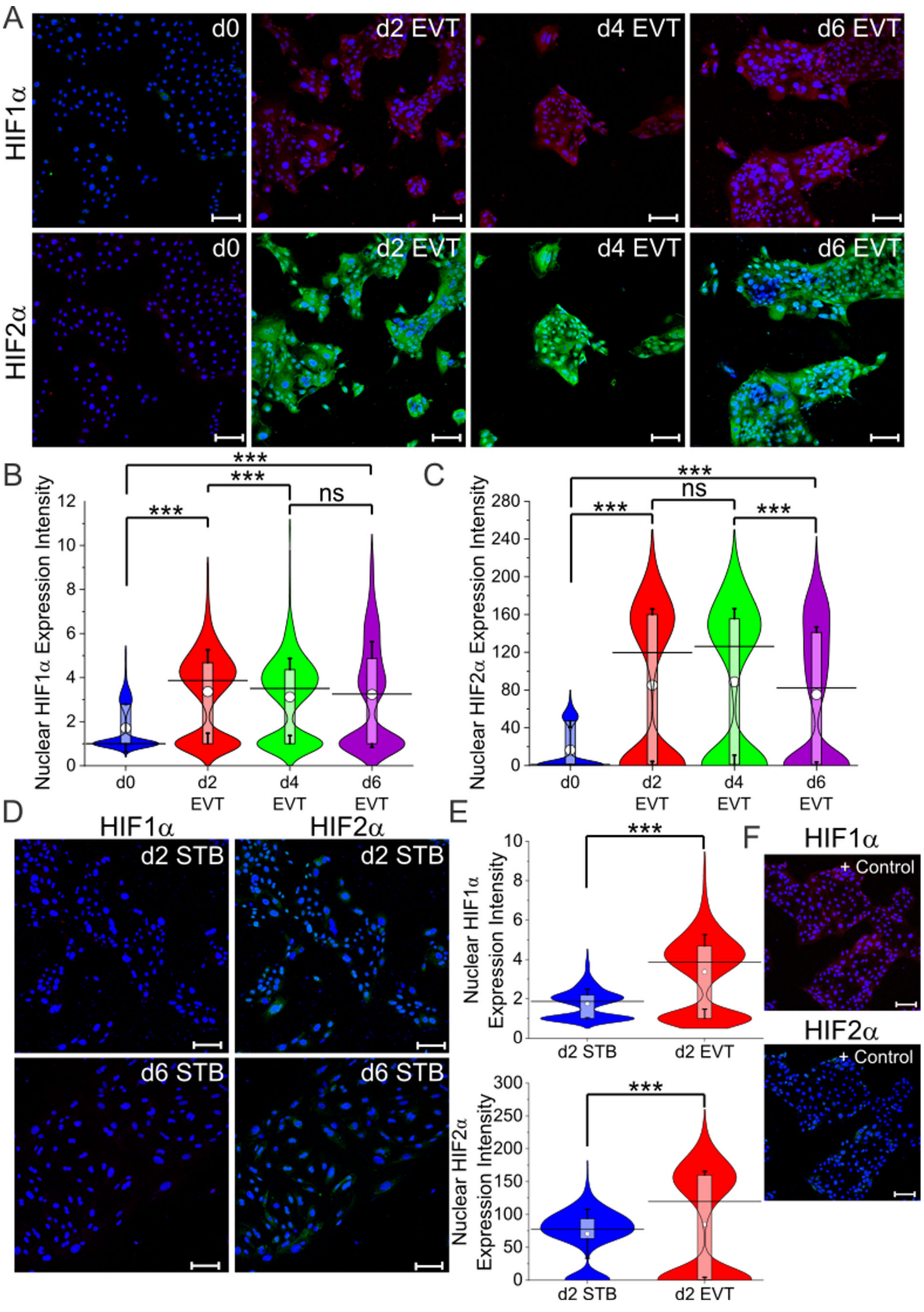
A
HIF1α
HIF2α
d0
d2 EVT
d4 EVT
d6 EVT
B
Nuclear HIF1α Expression Intensity
***
***
***
ns
d0
d2 EVT
d4 EVT
d6 EVT
C
Nuclear HIF2α Expression Intensity
***
ns
***
***
D
HIF1α
HIF2α
d2 STB
d6 STB
E
Nuclear HIF1α Expression Intensity
Nuclear HIF2α Expression Intensity
***
d2 STB
d2 EVT
F
HIF1α
HIF2α
+ Control

Figure S6. Expression of HIF1α and HIF2α is upregulated during EVT differentiation mediated by laminin-111.

(A) Confocal images of CT29 hTSCs on day 0, day 2, day 4, and day 6 of EVT differentiation, staining for HIF1α and HIF2α. Nuclei were stained with DAPI. Inset is the respective isotype control.

(B) Quantitative analysis of HIF1α expression intensity of CT29 hTSCs on day 0 (n=4001), day 2 (n=1131), day 4 (n=685), and day 6 (n=1453) of EVT differentiation. Analysis was performed in MATLAB and two biological replicates were used. The white circle represents the mean and the black bar represents the median. (ns, not significant, ***$p<0.0005$).

(C) Quantitative analysis of HIF2α expression intensity of CT29 hTSCs on day 0 (n=4001), day 2 (n=1131), day 4 (n=685), and day 6 (n=1453) of EVT differentiation. Analysis was performed in MATLAB and two biological replicates were used. The white circle represents the mean and the black bar represents the median. (ns, not significant, ***$p<0.0005$).

(D) Confocal images of CT29 hTSCs on day 2 and day 6 of STB differentiation, staining for HIF1α and HIF2α. Nuclei were stained with DAPI.

(E) Quantitative analysis of HIF1α and HIF2α expression intensity of CT29 hTSCs on day 2 of STB (n=555) and EVT (n=1131) differentiation. Analysis was performed in MATLAB and two biological replicates were used. The white circle represents the mean and the black bar represents the median. (***$p<0.0005$). Data for d2 EVT are the same as in panels B and C.

(F) Confocal images of CT29 hTSCs cultured in TSCM with the addition of 10 μM deferoxamine for two days, staining for HIF1α and HIF2α. Nuclei were stained with DAPI.

Scale bars are 100μm for all images.

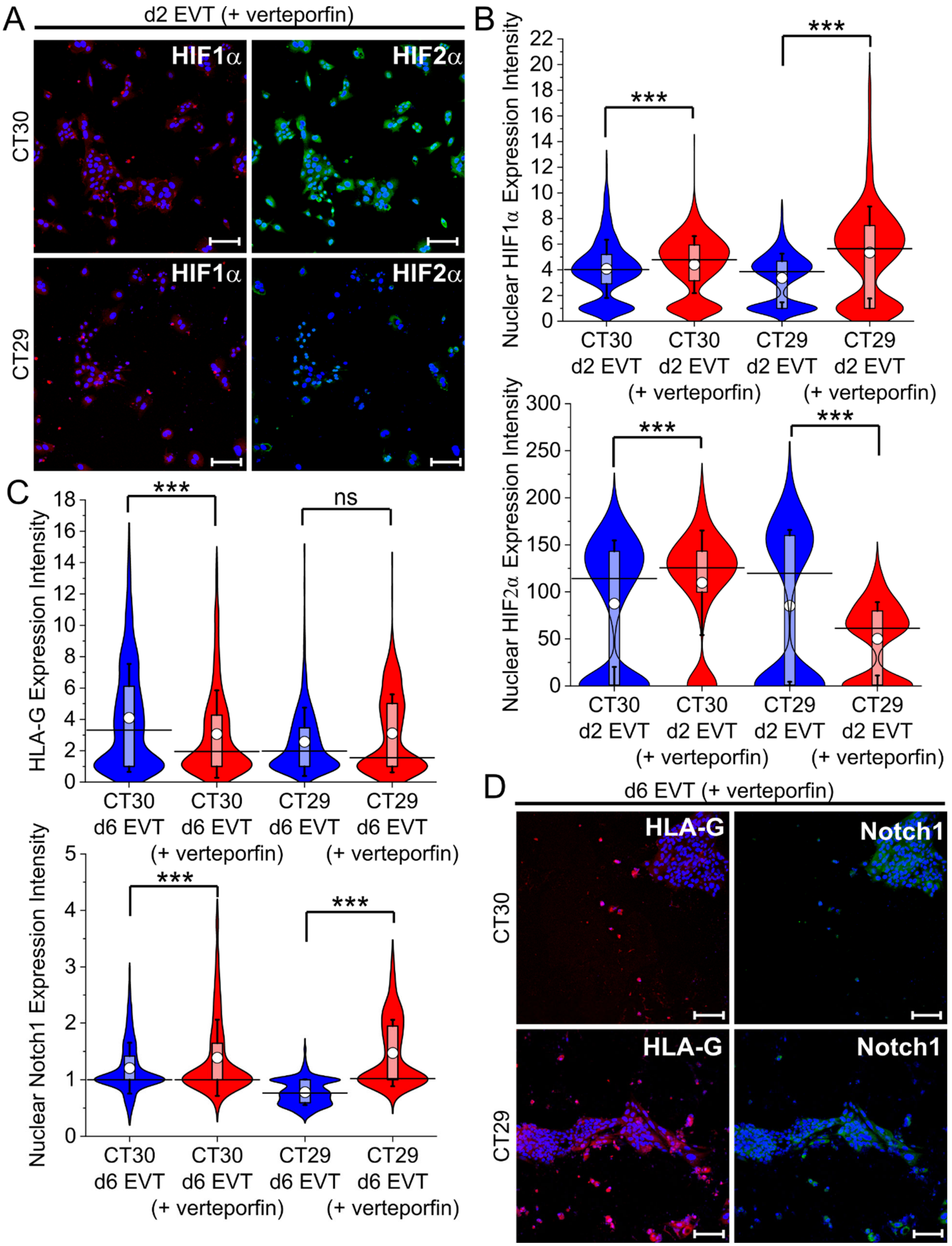

A
d2 EVT (+ verteporfin)
HIF1α
HIF2α
CT30
CT29
B
Nuclear HIF1α Expression Intensity
***
CT30 d2 EVT
CT30 d2 EVT (+ verteporfin)
CT29 d2 EVT
CT29 d2 EVT (+ verteporfin)
Nuclear HIF2α Expression Intensity
C
HLA-G Expression Intensity
ns
CT30 d6 EVT
CT30 d6 EVT (+ verteporfin)
CT29 d6 EVT
CT29 d6 EVT (+ verteporfin)
Nuclear Notch1 Expression Intensity
D
d6 EVT (+ verteporfin)
HLA-G
Notch1

Figure S7. Verteporfin does not significantly affect HIFα expression and commitment to EVT differentiation.

(A) Confocal images of CT30 and CT29 hTSCs on day 2 of EVT differentiation in the presence of the HIPPO-YAP inhibitor, verteporfin, staining for HIF1α and HIF2α. Nuclei were stained with DAPI.

(B) Quantitative analysis of HIF1α and HIF2α expression intensity of CT30 and CT29 hTSCs on day 2 of EVT differentiation in the absence (CT30: n=1591, CT29: n=1131) or presence (CT30: n=573, CT29: n=633) of the HIPPO-YAP inhibitor, verteporfin. Analysis was performed in MATLAB and two biological replicates were used. The white circle represents the mean and the black bar represents the median. (ns, not significant, ***p<0.0005). Data for EVT is same as used in Figs. 4 and S6.

(C) Quantitative analysis of HLA-G and Notch1 expression intensity of CT30 and CT29 hTSCs on day 6 of EVT differentiation in the absence (CT30: n=1309, HLA-G, n=1728, Notch1, CT29: n=729, HLA-G, n=1415, Notch1) or presence (CT30: n=578, CT29: n=343) of the HIPPO-YAP inhibitor, verteporfin. Analysis was performed in MATLAB and two biological replicates were used. The white circle represents the mean and the black bar represents the median. (ns, not significant, ***p<0.0005). Data for EVT is same as used in Figs. 3, S3 and S5.

(D) Confocal images of CT30 and CT29 hTSCs on day 6 of EVT differentiation in the presence of verteporfin, staining for HLA-G and Notch1. Nuclei were stained with DAPI.

Scale bars are 100μm for all images.

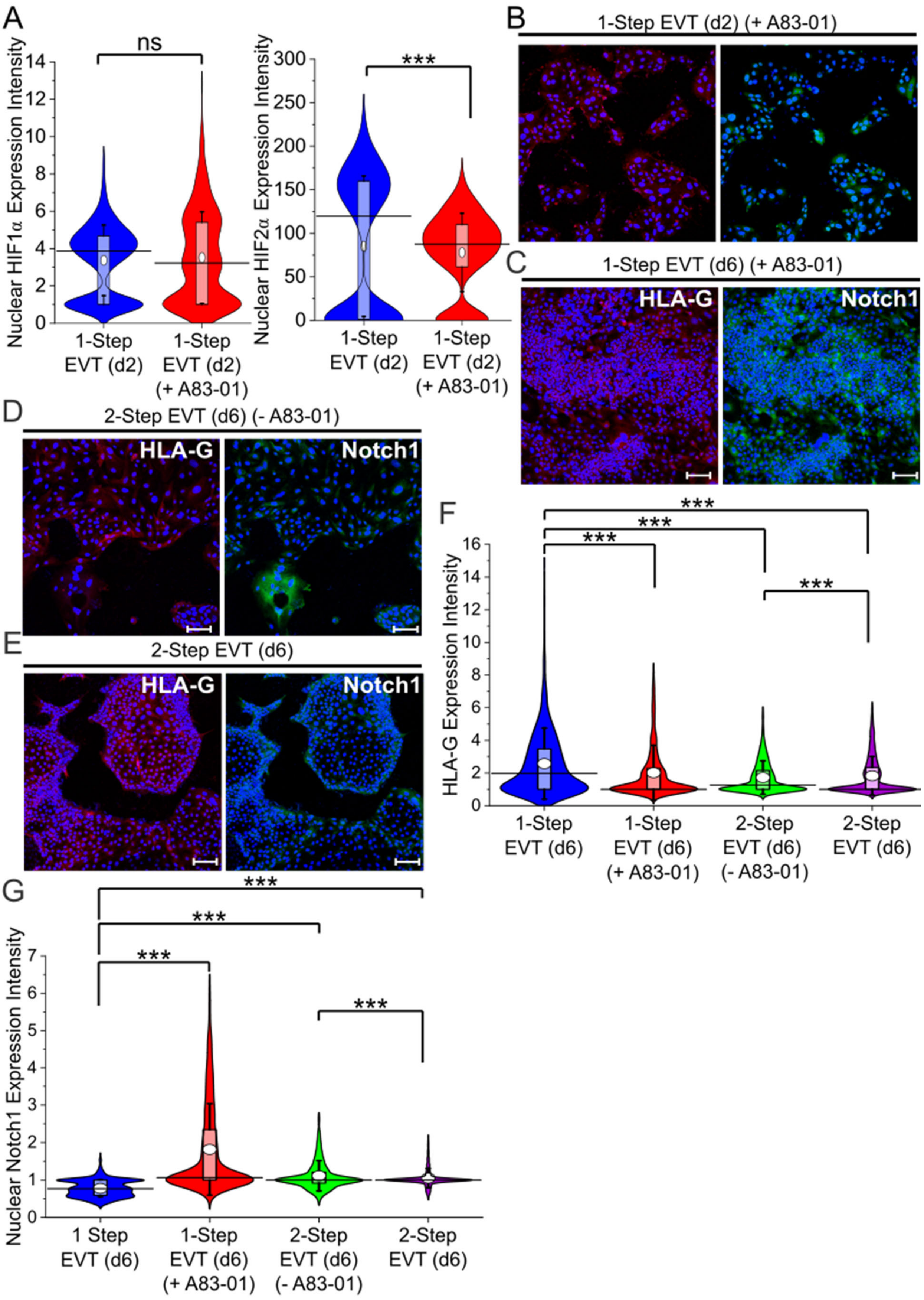
A
ns
Nuclear HIF1α Expression Intensity
14
12
10
8
6
4
2
0
1-Step EVT (d2)
1-Step EVT (d2) (+ A83-01)
***
Nuclear HIF2α Expression Intensity
300
250
200
150
100
50
0
1-Step EVT (d2)
1-Step EVT (d2) (+ A83-01)
B
1-Step EVT (d2) (+ A83-01)
C
1-Step EVT (d6) (+ A83-01)
HLA-G
Notch1
D
2-Step EVT (d6) (- A83-01)
HLA-G
Notch1
E
2-Step EVT (d6)
HLA-G
Notch1
F
***
***
***
***
HLA-G Expression Intensity
16
14
12
10
8
6
4
2
0
1-Step EVT (d6)
1-Step EVT (d6) (+ A83-01)
2-Step EVT (d6) (- A83-01)
2-Step EVT (d6)
G
***
***
***
***
Nuclear Notch1 Expression Intensity
7
6
5
4
3
2
1
0
1 Step EVT (d6)
1-Step EVT (d6) (+ A83-01)
2-Step EVT (d6) (- A83-01)
2-Step EVT (d6)

Figure S8: A defined system enables investigation of TGFβ signaling in EVT differentiation

(A) Quantitative analysis of HIF1α and HIF2α expression intensity of CT29 hTSCs on day 2 of EVT differentiation using the method described in Fig. 2A in the absence (labeled 1-Step, n=1131) or presence of the TGFβ inhibitor, A83-01 (n=645). Analysis was performed in MATLAB and two biological replicates were used. The white circle represents the mean and the black bar represents the median. (ns, not significant, ***p<0.0005). Data for d2 EVT is the same as used in Fig. S6 and S7.

(B) Confocal images of CT29 hTSCs on day 2 of EVT differentiation using the method described in Fig. 2A (1-Step) in the presence of the TGFβ inhibitor, A83-01, staining for HIF1α and HIF2α. Nuclei were stained with DAPI.

(C) Confocal image of CT29 hTSCs on day 6 of EVT differentiation using the method described in Fig. 2A (1-Step) in the presence of the TGFβ inhibitor, A83-01, staining for HLA-G and Notch1. Nuclei were stained with DAPI.

(D) Confocal image of CT29 hTSCs on day 6 of EVT differentiation using the method as previously described (32) (labeled 2-Step) in the absence of the TGFβ inhibitor, A83-01, staining for HLA-G and Notch1. Nuclei were stained with DAPI.

(E) Confocal image of CT29 hTSCs on day 6 of EVT differentiation using the method as previously described (32) (2-Step) which includes the TGFβ inhibitor, A83-01, staining for HLA-G and Notch1. Nuclei were stained with DAPI.

(F) Quantitative analysis of HLA-G expression intensity of CT29 hTSCs on day 6 of EVT differentiation using the method described in Fig. 2A in the absence (1-Step; n=729), or presence of the TGFβ inhibitor, A83-01 (n=5226), and the method as previously described (32) (2-Step) in the presence (n=7542) and absence (n=2596) of A83-01. Analysis was performed in MATLAB and two biological replicates were used. The white circle represents the mean and the black bar represents the median. (***p<0.0005). Data for 1-Step EVT in the absence of A83-01 is same as used in Fig. S3, S5 and S7.

(G) Quantitative analysis of Notch1 expression intensity of CT29 hTSCs on day 6 of EVT differentiation using the method described in Fig. 2A in the absence (1-Step; n=1415), or presence of the TGFβ inhibitor, A83-01 (n=5226), and the method as previously described (32) (2-Step) in the presence (n=7542) and absence (n=2596) of A83-01. Analysis was performed in MATLAB and two biological replicates were used. The white circle represents the mean and the black bar represents the median. (***p<0.0005). Data for 1-Step EVT in the absence of A83-01 is same as used in Fig. S3, S5 and S7.

Scale bars are 100μm for all images.

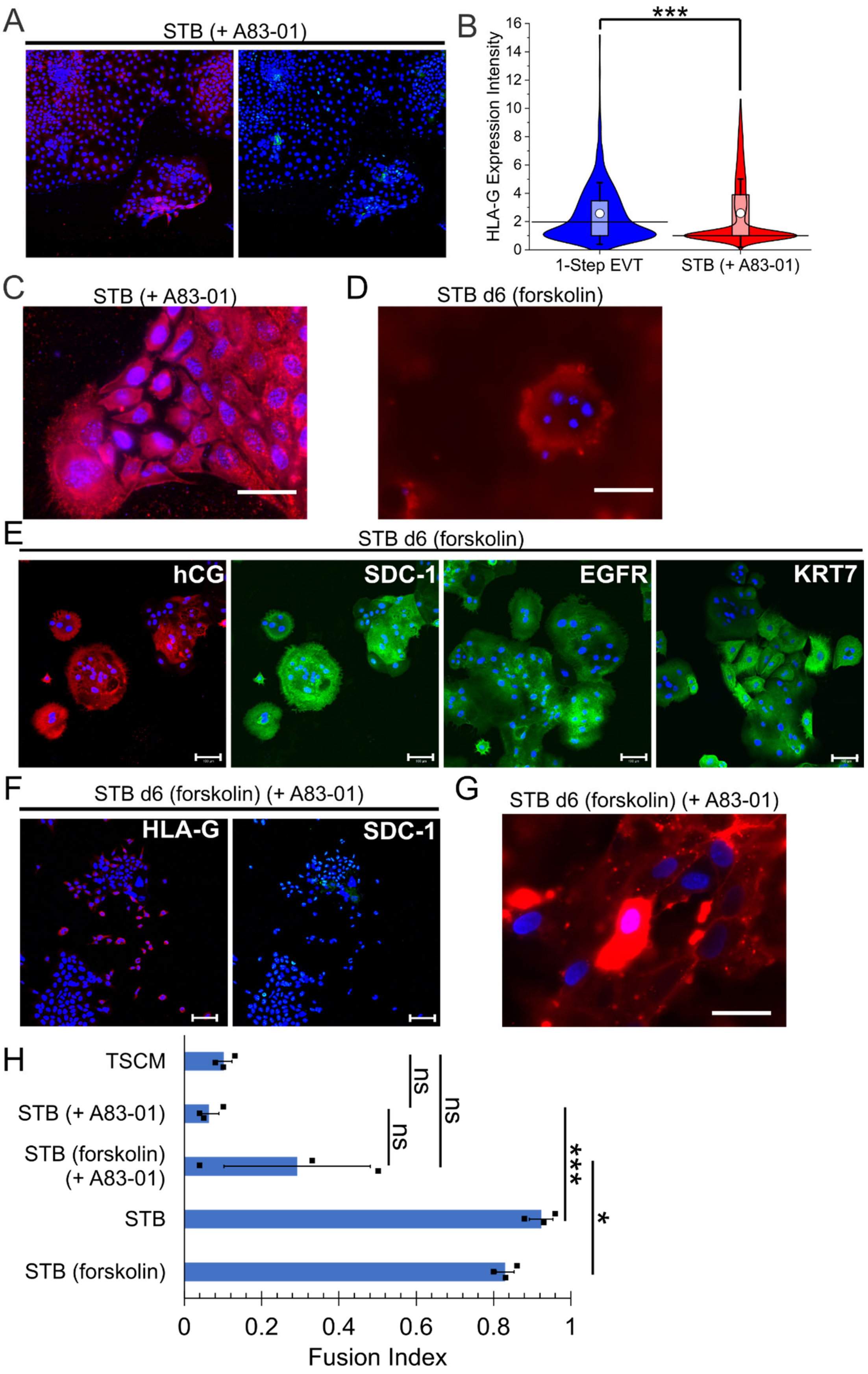

A
STB (+ A83-01)
B
HLA-G Expression Intensity
16
14
12
10
8
6
4
2
0
***
1-Step EVT
STB (+ A83-01)
C
STB (+ A83-01)
D
STB d6 (forskolin)
E
STB d6 (forskolin)
hCG
SDC-1
EGFR
KRT7
F
STB d6 (forskolin) (+ A83-01)
HLA-G
SDC-1
G
STB d6 (forskolin) (+ A83-01)
H
TSCM
STB (+ A83-01)
STB (forskolin)
(+ A83-01)
STB
STB (forskolin)
ns
ns
ns
***
*
0
0.2
0.4
0.6
0.8
1
Fusion Index

Figure S9: A defined system enables investigation of TGFβ signaling in STB differentiation.

(A) Confocal image of CT29 hTSCs on day 6 of STB differentiation using the method described in Fig. 1A in the presence of a A83-01, staining for HLA-G (left) and SDC-1 (right). Nuclei were stained with DAPI.

(B) Quantitative analysis of HLA-G expression intensity of CT29 hTSCs on day 6 of EVT differentiation using the method described in Fig. 2A (1-Step; n=729), or STB differentiation using the method described in Fig. 1A in the presence of a TGFβ inhibitor, A83-01 (n=10976). Analysis was performed in MATLAB and two biological replicates were used. The white circle represents the mean and the black bar represents the median. (***p<0.0005). Data for 1-Step EVT is same as used in Fig. S3, S5, S7, and S8.

(C) Fluorescent image of CT29 hTSCs on day 6 of STB differentiation using the method described in Fig. 1A in the presence of A83-01. Nuclei were stained with DAPI. Membrane was stained with Di-8-ANEPPS cell membrane stain. Scale bar is 50 μm.

(D) Fluorescent image of CT29 hTSCs on day 6 of STB differentiation using the method using forskolin as previously described (32). Nuclei were stained with DAPI. Membrane was stained with Di-8-ANEPPS cell membrane stain. Scale bar is 50 μm.

(E) Confocal images of CT29 hTSCs on day 6 of STB differentiation using the method using forskolin as previously described (32), staining for hCG, SDC-1, EGFR, and KRT7. Nuclei were stained with DAPI.

(F) Confocal images of CT29 hTSCs on day 6 of STB differentiation using the method using forskolin as previously described (32) in the presence of the TGFβ inhibitor, A83-01, staining for HLA-G and SDC-1. Nuclei were stained with DAPI.

(G) Fluorescent image of CT29 hTSCs on day 6 of STB differentiation using the method using forskolin as previously described (32) in the presence of the TGFβ inhibitor, A83-01. Nuclei were stained with DAPI. Membrane was stained with Di-8-ANEPPS cell membrane stain. Scale bar is 50 μm.

(H) Fusion efficiency of CT29 hTSCs on day 6 of STB differentiation using the method described in Fig. 1A and the method using forskolin as previously described (32), in the presence and absence of the TGFβ inhibitor, A83-01, compared to CT29 hTSCs cultured in TSCM. Nuclei were stained with DAPI. Membrane was stained with Di-8-ANEPPS cell membrane stain. Three measurements from two biological replicates were used to calculate fusion index. Data for TSCM, STB, and STB (forskolin) is same as used in Fig. S1. (ns, not significant, *p<0.05, ***p<0.0005, Error bars, S.D., n=3).

Scale bars are 100μm for all images unless specified otherwise.

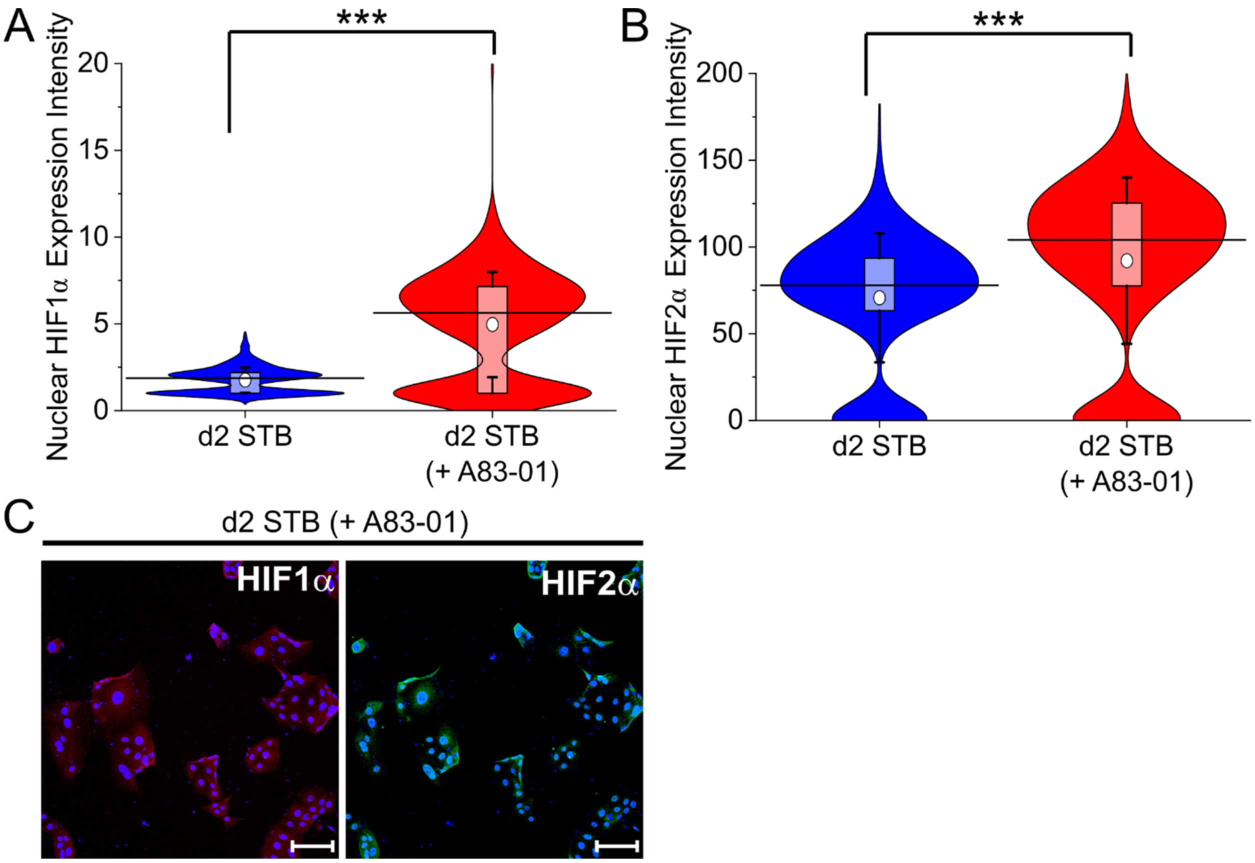

Figure S10: TGFβ inhibition upregulates HIF1α.

(A) Quantitative analysis of HIF1α expression intensity of CT29 hTSCs on day 2 of STB differentiation using the method described in Fig. 1A in the absence (n=555) or presence (n=752) of a TGFβ inhibitor, A83-01. Analysis was performed in MATLAB and two biological replicates were used. The white circle represents the mean and the black bar represents the median. (***$p<0.0005$). Data for d2 STB are the same as used in Fig. S6.

(B) Quantitative analysis of HIF2α expression intensity of CT29 hTSCs on day 2 of STB differentiation using the method described in Fig. 1A in the absence (n=555) or presence (n=752) of a TGFβ inhibitor, A83-01. Analysis was performed in MATLAB and two biological replicates were used. The white circle represents the mean and the black bar represents the median. (***$p<0.0005$). Data for d2 STB are the same as used in Fig. S6.

(C) Confocal images of CT29 hTSCs on day 2 of STB differentiation using the method described in Fig. 1A in the presence of a A83-01, staining for HIF1α and HIF2α. Nuclei were stained with DAPI.

Scale bars are 100μm for all images.
